# Robotic-assisted benign hysterectomy compared with laparoscopic, vaginal, and open surgery: a systematic review and meta-analysis

**DOI:** 10.1007/s11701-023-01724-6

**Published:** 2023-10-19

**Authors:** Louis Lenfant, Geoffroy Canlorbe, Jérémie Belghiti, Usha Seshadri Kreaden, April E. Hebert, Marianne Nikpayam, Catherine Uzan, Henri Azaïs

**Affiliations:** 1 Department of Urology, Academic Hospital Pitié-Salpêtrière, APHP, Sorbonne Université, 75013 Paris, France; 2 Department of Surgery and Oncological Gynecology, Pitié-Salpétrière University Hospital, Assistance Publique des Hôpitaux de Paris, Sorbonne University, Paris, France; 3 Biostatistics & Global Evidence Management, Intuitive Surgical Inc, Sunnyvale, CA USA; 4 Gynecologic and Breast Oncologic Surgery Department, Georges Pompidou European Hospital, APHP, Centre, Université de Paris Cité, Paris, France

**Keywords:** Benign uterine pathology, Hysterectomy, Laparoscopic hysterectomy, Robotic surgery, Open hysterectomy, Vaginal hysterectomy

## Abstract

The potential benefits and limitations of benign hysterectomy surgical approaches are still debated. We aimed at evaluating any differences with a systematic review and meta-analysis. PubMed, MEDLINE, and EMBASE databases were last searched on 6/2/2021 to identify English randomized controlled trials (RCTs), prospective cohort and retrospective independent database studies published between Jan 1, 2010 and Dec 31, 2020 reporting perioperative outcomes following robotic hysterectomy versus laparoscopic, open, or vaginal approach (PROSPERO #CRD42022352718). Twenty-four articles were included that reported on 110,306 robotic, 262,715 laparoscopic, 189,237 vaginal, and 554,407 open patients. The robotic approach was associated with a shorter hospital stay (*p* < 0.00001), less blood loss (*p* = 0.009), and fewer complications (OR: 0.42 [0.27, 0.66], *p* = 0.0001) when compared to the open approach. The main benefit compared to the laparoscopic and vaginal approaches was a shorter hospital (R/L WMD: – 0.144 [– 0.21, – 0.08], *p* < 0.0001; R/V WMD: – 0.39 [– 0.70, – 0.08], *p* = 0.01). Other benefits seen were sensitive to the inclusion of database studies. Study type differences in outcomes, a lack of RCTs for robotic vs. open comparisons, learning curve issues, and limited robotic vs. vaginal publications are limitations. While the robotic approach was mainly comparable to the laparoscopic approach, this meta-analysis confirms the classic benefits of minimally invasive surgery when comparing robotic hysterectomy to open surgery. We also reported the advantages of robotic surgery over vaginal surgery in a patient population with a higher incidence of large uterus and prior surgery.

**Supplementary Information:**

The online version contains supplementary material available at 10.1007/s11701-023-01724-6.

## Introduction

Hysterectomy for benign uterine pathology is one of the most common surgical procedures, with half a million cases each year in the United States [1] and nearly 42,000 cases each year in France [2]. Historically, hysterectomy was performed via an open or vaginal approach, but a shift to laparoscopy was made in the nineties. During the past 2 decades, robotic-assisted laparoscopic hysterectomy has been popularized as a standard minimally invasive procedure for the treatment of benign uterine pathology. The robotic platform allows for a magnified 3D view and improved manipulation using fully wristed instruments, thus improving the ergonomics of laparoscopic surgery, which was the inherent drawback of standard straight-stick laparoscopy. Currently, an estimated 65% of hysterectomies in the USA are performed using a minimally invasive approach, [1] with up to 43% of laparoscopic surgeries done with robotic assistance [3]. In France, of the 42,000 benign hysterectomies performed in 2019, 43% were performed via laparoscopy versus 25% via the open approach [2, 4].

However, head-to-head comparisons involving all surgical approaches are lacking.

Previous meta-analyses have compared the robotic approach to either the open [5] and vaginal route or to the laparoscopic route [6–8]. When compared with open or vaginal approach, the robotic approach may offer a shorter length of hospital stay and recovery period, as well as less blood loss [5]. Yet, there is contradictory evidence to support the robotic versus the laparoscopic approach. Four reviews reported similar perioperative outcomes between the robotic technique and laparoscopy [5, 9–11]. Moreover, the same reviews reported significantly higher costs associated with the use of the robotic platform (1.5–3 times higher). Lastly, most reviews included retrospective studies of low levels of evidence. Thus, the role of robotic surgery within the surgical armamentarium for benign hysterectomy remains unclear.

Against this backdrop, we aimed to provide a comprehensive and updated systematic review and meta-analysis of the available evidence to compare perioperative outcomes of the robotic approach to other existing surgical approaches to treat benign uterine pathology.

## Materials and methods

### Search strategy

Systematic searches of PubMed, SCOPUS, and EMBASE were conducted, with the last search performed on 6/2/2021. This was done using a free-text protocol using combinations of robotic terms, indication terms, anatomic terms, and procedure terms. The detailed search methodology is depicted in Online Resource 1. Searches were limited to English language and publication date between Jan 1, 2010 (after the da Vinci Si Surgical System and the later Xi and X systems became widely available) and Dec 31, 2020. This systematic review was registered on PROSPERO (registration number CRD42022352718), but no separate protocol was prepared. Duplicates were removed, and reports were independently reviewed by two authors (A.H. and U.K.).

### Inclusion and exclusion criteria

Based on the Preferred Reporting Items for Systematic Reviews and Meta-analyses (PRISMA) statement [12], we used the Patient-Intervention-Comparison-Outcome-Study design (PICOS) approach to further select RCTs, prospective comparative studies, and independent database studies (*n* ≥ 20 for each cohort) in which robotic cases were compared to laparoscopic, open, or vaginal cases. Exclusion criteria were met if: (1) the study was not in English, (2) the paper reported on a pediatric population, (3) the article was a health technology assessment (HTA) not published in a peer-reviewed journal, (4) the surgery involved alternative surgical techniques (i.e. single-port), (5) data for each surgical approach was not provided separately, (6) there was no analysis stratified by benign hysterectomy (i.e. benign data mixed with cancer data, or hysterectomy data mixed with other procedures), (7) there were no outcomes of interest (conversions, intraoperative complications, blood transfusions and/or estimated blood loss, operative time, postoperative complications, length of hospital stay (LOS), readmissions, mortality), or (8) the study included patient populations already included. Any disagreement regarding study inclusion was resolved by discussion with H.A., L.L., and G.C.

### Data extraction

Data were independently extracted from all included studies by two authors (A.H and U.K) with subsequent cross-checks to ensure their accuracy. Study-level data included the citation, study design, study period, country, and the overall number of patients included, as well as other information to determine if the paper met inclusion and exclusion criteria. Patient-level data included age, body mass index (BMI), uterine weight, proportion of uterus defined as large (i.e., > 200–250 g) [13], and the existence of a surgical antecedent. Treatment-level data included the surgical approach and the sample size of each intervention group. Peri-operative outcomes included the operative time, length of hospital stay (LOS), estimated blood loss (EBL), intraoperative complications, and per-operative conversion. Post-operative safety outcomes included complications, readmissions, reoperations, and mortality within 30 days of surgery.

### Risk of bias assessment

Two reviewers (A.H and U.K) independently evaluated the included studies using the Cochrane Handbook risk of bias tools for randomized trials (RoB-2) [14], and the Newcastle Ottawa Score (NOS) for independent database and non-randomized prospective comparison studies [15]. All disagreements were resolved through discussion (A.H, U.K, H.A, L.L and G.C).

### Data and statistical analyses

Descriptive statistics were used to summarize extracted baseline patient characteristics. Data were displayed in the form of forest plots and pooled analysis was performed whenever at least two studies contained sufficient data. A weighted mean difference and 95% confidence interval (WMD, 95% CI) were calculated for continuous measures using the inverse variance method. An odds ratio with 95% confidence interval (OR, 95% CI) was calculated for discrete measures using the Mantel–Haenszel (M–H) method, except in the case of at least 2 papers reported zero events for both cohorts, in which case, a risk difference (RD, 95% CI) was calculated. A random effects model was used for statistically significant heterogeneity (Chi^2^ *p* < 0.05, *I*^2^ ≥ 50%). A *p*-value of < 0.05 was considered statistically significant. For each forest plot, papers were stratified according to study type. Sensitivity analyses were performed as deemed necessary. All calculations, Forest Plots, and Funnel Plots were done using Review Manager (Version 5.3. Copenhagen: The Nordic Cochrane Centre, The Cochrane Collaboration, 2014) and continuous data not reported as mean (SD) were converted using Review Manager calculators when possible. Funnel plots were created to test for publication bias when there were at least ten available studies (as recommended by Cochrane).

## Results

### Systematic review

**Fig. 1:**
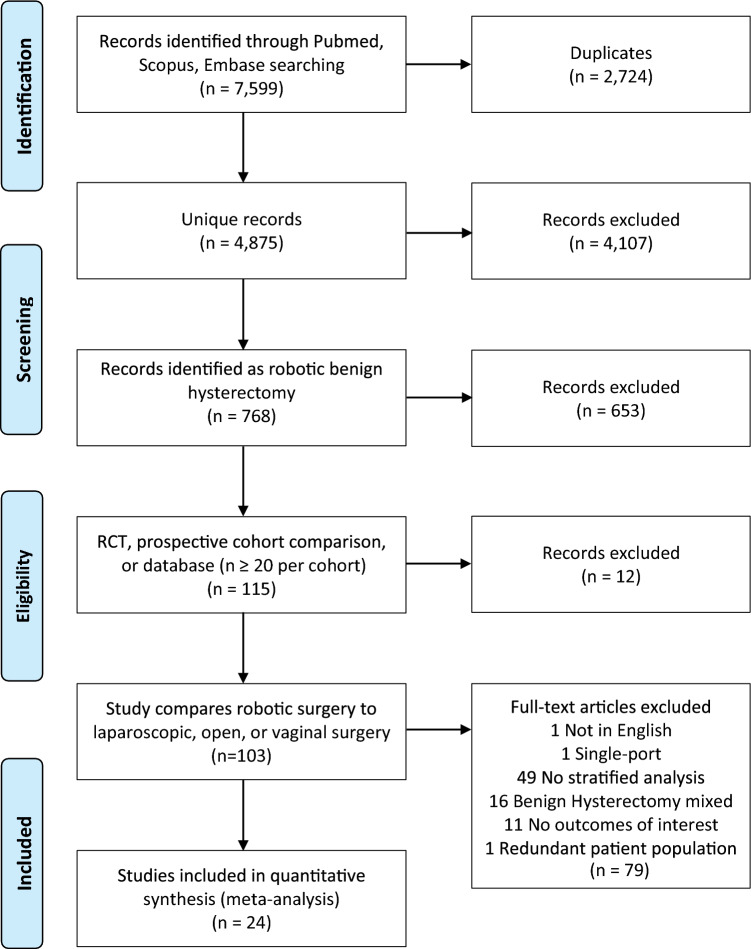
Flowchart accounting for each paper found in the initial searches, from identification through screening, showing the final number of articles included in the meta-analysis and the reasons for exclusion. *RCT* randomized controlled trial

There were 1635 references identified in the Pubmed search, 3926 in the Scopus search, and 2038 in the Embase search. Duplicates were removed, leaving 4875 unique references (Online Resource 2). Based on the aforementioned inclusion and exclusion criteria, a final cross-checked selection was made of 24 studies published between 2010 and 2020, as presented in the PRISMA flowchart in Fig. 1.

### Overall characteristics of included studies

Of the 24 studies included in the pooled analysis [16–39], 4 were RCTs, all of which compared robotic to laparoscopic and/or vaginal cases, 5 were prospective studies (one quasi-RCT), and 15 were independent database articles. The sample size in the overall pooled population was 1,116,665, of whom 110,306 underwent robotic hysterectomy, 554,407 underwent open hysterectomy, 189,237 had a vaginal procedure, and 262,715 were operated on using a conventional laparoscopic approach. Overall, 22, 11, and 10 studies compared the robotic approach to the laparoscopic, open and vaginal approach, respectively (Table 1).

**Table 1:** Characteristics of included studies

Author	Country	Study design	Study arms	Sample size	Time period	Follow-up
Billfeldt et al. (2018) [16]	Sweden	Database	Robotic	1015	2009–2015	1 year
			Lap	1539		
			Vaginal	3767		
			Open	7485		
Brunes et al. (2021) [17]	Sweden	Database	Robotic	249	Jan. 1, 2015–Dec. 31, 2017	1 year
			Lap	317		
			Vaginal	218		
			Open	751		
Carbonnel et al. (2013) [18]	France	Pro Compare	Robotic	60	Mar. 2010–Mar. 2012	2 months
			Vaginal	34		
Cohen et al. (2014) [19]	USA	Database	Robotic Benign	18,836	2009	Discharge
			Lap Benign	84,569		
			Vaginal Benign	77,940		
			Open Benign	232,633		
Dandolu et al. (2018) [20]^a^	USA	Database	Robotic	12,029	2008–2012	90 days
			Lap	15,971		
			Vaginal	43,936		
			Open	145,047		
Deimling et al. (2017) [21]	USA	RCT	Robotic	72	Apr. 23–Oct. 20, 2014	3 months
			Lap	72		
Dubeshter et al. (2013) [22]^b^	USA	Database	Robotic	1192	2011	Discharge
			Lap	2557		
			Vaginal	2200		
			Open	7926		
Elessawy et al. (2020) [23]	Germany	Pro Compare	Robotic	56	Jan. 1, 2013–Dec. 31, 2017	20 weeks
			Lap	99		
Friedman et al. (2016) [24]	USA	Database	Inpatient Matched		2011	1 month
			Robotic	7056		
			Non-Robotic	7056		
			Outpatient Matched			
			Robotic	7199		
			Non-Robotic	7199		
			Inpatient Matched			
Hart et al. (2013) [25]^c^	USA	Database	Robotic	3971	Jan. 1, 2009–Jun. 30, 2011	Discharge
			Lap	4363		
			Endo Stitch	974		
Herrinton et al. (2020) [26]	USA	Database	Robotic	560	Jan. 1, 2011–Sept. 30, 2015	90 days
			Lap	6785		
Lim et al. (2016) [27] Risk	USA	Database	Robotic	4528	Jan. 1, 2013–Jul. 2, 2014	1 month
			Lap	2464		
Lim et al. (2016) [28] Multi	USA	Database	Robotic	2300	Jan. 1, 2010–Sept. 30, 2013	1 month
			Lap	11,952		
			Vaginal	8121		
			Open	9745		
Lonnerfors et al. (2015) [29]^d^	Sweden	RCT	Robotic	61	Jan. 2010–Jun. 2013	4 months
			Lap	36		
			Vaginal	25		
Luciano et al. (2016) [30]	USA	Database	Robotic	20,781	Jan. 2005–Dec. 2010	1 month
			Lap	78,148		
			Vaginal	52,635		
			Open	138,311		
Martinez-Maestre et al. (2014) [31]	Spain	Pro Compare	Robotic	51	Jan. 2008–Dec. 2009	1 month
			Lap	54		
Ngan et al. (2018) [32]	Canada	Database	Robotic	10,677	2008–2012	Discharge
			Lap	33,088		
Paraiso et al. (2013) [33]	USA	RCT	Robotic	26	June.2007–Mar. 2011	6 months
			Lap	26		
Pellegrino et al. (2017) [34]	Italy	Pro Compare	Robotic	64	Sept. 2014–Sept. 2015	3 months
			Lap	130		
			Open	74		
Rosero et al. (2013) [35]	USA	Database	Robotic	7788	2009–2010	Discharge
			Lap	7788		
Sarlos et al. (2012) [36]	Switzerland	RCT	Robotic	47	2008–2011	2 months
			Lap	48		
Swenson et al. (2016) [37]^e^	USA	Database	Robotic	1338	Jan. 1, 2013–Jul. 1, 2014	1 month
			Lap	539		
			Vaginal	361		
Ulubay et al. (2016) [38]	Turkey	Pro Compare	Robotic	20	Jan. 2011–Jan. 2015	6 months
			Open	20		
Wright et al. (2013) [39]^f^	USA	Database	Robotic	4971	2007–2010	1 month
			Lap	4971		
			Robotic	5359		
			Open	5359		

*Lap* laparoscopic, *pro* prospective, *RCT* randomized controlled trial

^a^Sample size is for outcomes, for demographic characteristic, sample size is: Robotic (not reported), Laparoscopic 28,871, Vaginal 50,222, and Open 165,752 (also reports laparoscopic-assisted supracervical and laparoscopic-assisted vaginal groups not included in analysis)

^b^Sample size is for all benign indications combined

^c^Reports an Endo Stich laparoscopic group not included in the analysis

^d^Randomization was done on the basis of robotic versus non-robotic minimally invasive (laparoscopic and vaginal)

^e^Reports a laparoscopic-assisted vaginal group not included in the analysis

^f^Sample size is matched, unmatched sample sizes are: Robotic 10,797, Laparoscopic 75,761, Vaginal 54,912, Open 123,288

### Risk of bias assessment

**Fig. 2:**
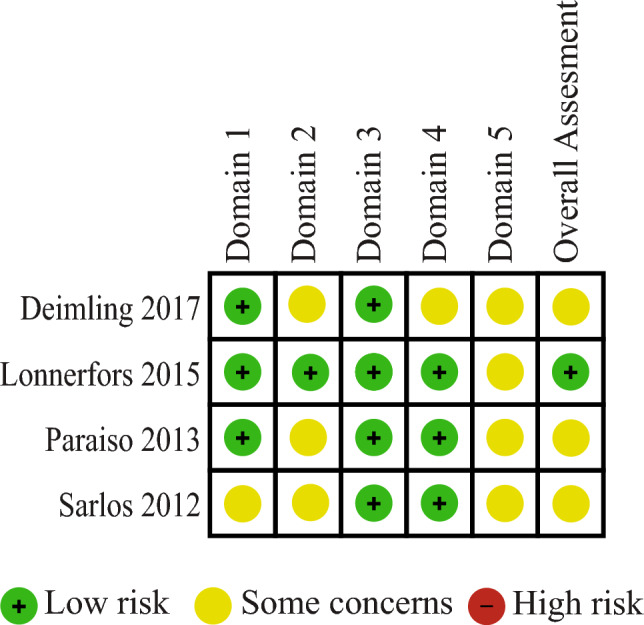
Risk of bias assessments for randomized controlled trials using the Cochrane Handbook risk of bias tools for randomized trials (RoB-2). Domain 1 deals with bias arising from the randomization process, Domain 2 deals with bias due to deviations from the intended interventions, Domain 3 deals with bias due to missing outcome data, Domain 4 deals with bias in measurement of the outcome, and Domain 5 deals with bias in selection of the reported result

Risk of bias (RoB) assessments for the four RCTs were low regarding randomization, missing data, and outcomes measurements (Fig. 2). However, due to some concerns regarding deviations from the intended intervention, as well as the selection of reported results, the overall ROBs of three out of the four RCTs were considered as having some concerns.

For the database and prospective cohort studies, the Newcastle–Ottawa scores ranged between 6 and 9 for the included cohort studies, with a lack of specifying whether patients were lost to follow-up being the most common reason for a lower score (Online Resource 3).

### Baseline patient characteristics

Results for the meta-analysis of baseline patient characteristics are reported in Table 2.

**Table 2:** Baseline patient characteristics

Meta-analysis						Heterogeneity		
Outcome	# Studies	Robot n	Comparator n	Effect size [95% CI]	*p*-value	Model	*I*^2^	*p*-value
Robotic vs. Laparoscopic								
Age (year)	**14**	**56,406**	**190,162**	**MD: 0.95 [0.18, 1.71]**	**0.01**	**RE**	**98%**	** < 0.00001**
BMI	8	1715	1803	MD: 0.25 [– 0.64, 1.14]	0.58	**RE**	**67%**	**0.003**
Uterine weight (g)	**6**	**1579**	**802**	**MD: – 23.6 [– 33.45, – 13.91]**	** < 0.0001**	FE	0%	0.88
# Large uterus	3	2842	18,362	OR: 1.10 [1.00, 1.22]	0.06	FE	30%	0.24
Prior surgery	7	2623	3214	OR: 0.97 [0.74, 1.25]	0.79	**RE**	**61%**	**0.02**
Robotic vs. Open								
Age	**7**	**71,887**	**554,020**	**MD: 0.77 [0.20, 1.33]**	**0.008**	**RE**	**99%**	** < 0.00001**
BMI	2	84	94	MD: – 1.14 [– 5.05, 2.77]	0.57	**RE**	**91%**	**0.0007**
# Large uterus	2	3315	17,230	OR: 1.47 [0.14, 15.45]	0.75	**RE**	**100%**	** < 0.00001**
Prior surgery	2	84	94	OR: 0.37 [0.03, 5.39]	0.47	**RE**	**81%**	**0.02**
Robotic vs. Vaginal								
Age (year)	6	71,863	192,719	MD: – 1.74 [– 3.64, 0.16]	0.07	**RE**	**100%**	** < 0.00001**
Uterine weight (g)	3	1459	420	MD: 0.70 [– 126.49, 127.90]	0.99	**RE**	**99%**	** < 0.00001**
# Large uterus	**2**	**3315**	**11,888**	**OR: 1.87 [1.13, 3.11]**	**0.02**	**RE**	**95%**	** < 0.00001**
Prior surgery	**2**	**1075**	**3801**	**OR: 1.79 [1.56, 2.05]**	** < 0.00001**	FE	0%	0.75

*CI* confidence interval, *MD* mean difference, *BMI* body mass index, *OR* odds ratio, *RD* risk difference, *RE* random effects model, *FE* fixed effects model

Bolding indicates significance (*p* < 0.05)

### Robotic versus laparoscopic approach

Patients who underwent robotic hysterectomy were significantly older than those operated via the laparoscopic approach, with a mean difference of 0.95 years (*p* = 0.01). The mean uterine weight was higher for laparoscopic patients, with a mean difference of 23 g (*p* < 0.0001) compared to robotic patients. The proportion of large uteri (i.e., uterine weight > 200–250 g) was higher in the robotic group (OR: 1.10 [1.00, 1.22]) but this difference was not significant (*p* = 0.06). BMI and the proportion of patients who had prior surgery was not different between groups.

### Robotics versus open approach

Patients operated on by robotics were older than those operated on by the open route, with a mean difference of 0.77 years (*p* = 0.008). There were no differences in BMI, proportion of large uterus, or previous surgery.

### Robotics versus vaginal approach

There were no statistical differences in term of age or uterus weight between the robotic and the vaginal approach. However, the proportion of large uteruses (uterus weight > 250 g/8 weeks) was higher in the robotic group (623/3315, 18.8% vs. 1305/11888, 11%, *p* = 0.02). There was also a significantly higher rate of previous surgery in the robotic group (518/1075, 48% vs. 1333/3801, 35%, *p* < 0.00001).

### Peri-operative outcomes

#### Robotic versus laparoscopic approach

**Fig. 3:**
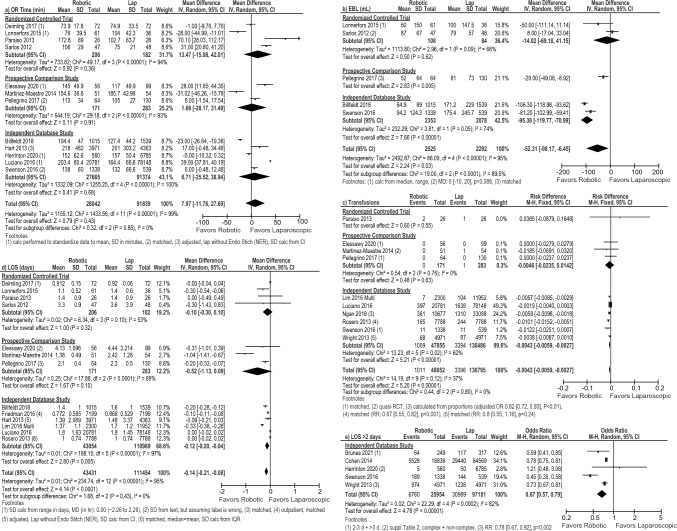
Forest plots for the robotic versus laparoscopic comparison showing the results of the overall pooled results and the study type subgroup analyses for **a** operative (OR) time in minutes (min), **b** estimated blood loss (EBL) in milliliters (mL), **c** blood transfusions, **d** length of hospital stay (LOS) in days, and **e** the number of patients requiring a hospital stay greater than 2 days. *Lap* laparoscopic, *SD* standard deviation, *IV* inverse variance, *CI* confidence interval, *calc* calculation, *MD* mean difference, *hr* hour, *IQR* interquartile range, *OR* odds ratio, *RR* risk ratio

Individual meta-analyses of four RCTs [21, 29, 33, 36], three prospective comparison studies [23, 31, 34] and five independent database studies [16, 25, 26, 30, 37], and the overall pooled analysis showed no difference in operative time (Fig. 3a).

The overall pooled estimated blood loss (EBL) was less after the robotic versus the laparoscopic approach (MD: – 52.31 mL [– 98.17, – 6.45], *I*^2^ = 95%, *p* = 0.03); however, the sub-analysis of RCTs [29, 36] was not significant (MD: – 14.02 mL [– 69.18, 41.15], *I*^2^ = 66%, *p* = 0.62 Fig. 3b). This same pattern was seen with blood transfusions; the overall pooled estimate favored robotic (RD: – 0.0043 [– 0.0059, – 0.0027], *I*^2^ = 37%, *p* < 0.00001), but the one RCT (RD: 0.0385 [– 0.0879, 0.1648], *p* = 0.55) and the prospective subgroup analysis were not significant (RD: – 0.0046 [– 0.0235, 0.0142], *I*^2^ = 0%, *p* = 0.63, Fig. 3c).

While the subgroup meta-analysis of four RCTs [21, 29, 33, 36] and the subgroup meta-analysis of three prospective studies [23, 31, 34] showed no difference in average LOS between the robotic and the laparoscopic approach (RCT MD: – 0.10 days [– 0.30, 0.10], *I*^2^ = 53%, *p* = 0.32; Prospective MD: – 0.52 [– 1.13, 0.09], *I*^2^ = 89%, *p* = 0.10, Fig. 3d), the meta-analysis of independent database studies (MD: – 0.12 days [– 0.20, – 0.04], *I*^2^ = 97%, *p* = 0.005) and the overall pooled result (MD: – 0.14 days [– 0.21, – 0.08], *I*^2^ = 95%, *p* < 0.0001) were both significant, but with a robotic benefit of only 3 h.

When measured as the proportion of patients requiring a hospital stay greater than 2 days, the meta-analysis (all database studies) showed that patients had a lower risk of staying more than 2 days post-operatively after a robotic procedure compared to the laparoscopic approach (OR: 0.67 [0.57, 0.79], *I*^2^ = 82%, *p* < 0.0001, Fig. 3e).

**Fig. 4:**
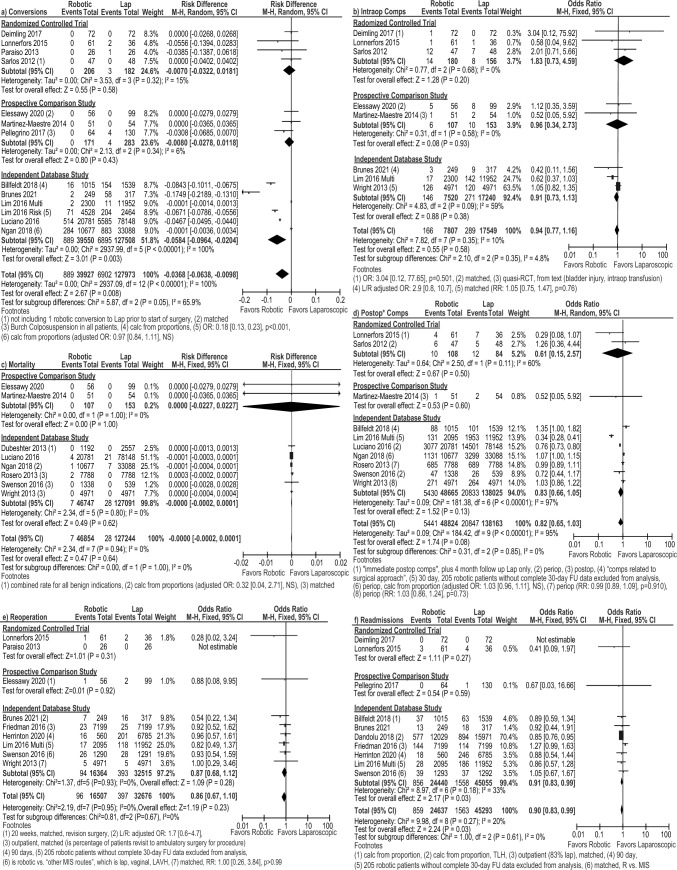
Forest plots for the robotic versus laparoscopic comparison showing the results of the overall pooled results and the study type subgroup analyses for **a** conversions to open surgery, **b** intraoperative complications (Intraop Comps), **c** mortality, **d** postoperative complications (Postop* Comps), **e** reoperations, and **f** readmissions. *L or Lap* laparoscopic, *M–H* Mantel–Haenszel, *CI* confidence interval, *calc* calculated, *OR* odds ratio, *adj* adjusted, *NS* not significant, *RCT* randomized controlled trial, *R* robotic, *RR* risk ratio, periop = perioperative, *FU* follow-up, *LAVH* laparoscopic-assisted vaginal hysterectomy, *MIS* minimally invasive surgery, *TLH* total laparoscopic hysterectomy. *Indicates that the postoperative complication rate within 30 days was extracted preferentially, but if the paper only reported a perioperative rate, it was included so as to not exclude that paper from the analysis

Conversion rates were similar between both groups in the subgroup meta-analysis of four RCTs [21, 29, 33, 36] and three prospective studies [23, 31, 34]. The subgroup meta-analysis of database studies showed a lower risk of conversion for the robotic approach (RD: – 0.06 [– 0.10, – 0.02], *I*^2^ = 100%, *p* = 0.003), as did the overall pooled analysis (RD: – 0.04 [– 0.06, – 0.01], *I*^2^ = 100%, *p* = 0.008, Fig. 4a). There were no significant differences in intraoperative or postoperative complications, mortality, or reoperations between the robotic approach and the laparoscopic approach (Fig. 4b–e**)**. Readmissions was significant in favor of the robotic group for the overall pooled analysis (OR: 0.90 [0.83, 0.99], *I*^2^ = 20%, *p* = 0.03), which was driven by the database subgroup (OR: 0.91 [0.83, 0.99], *I*^2^ = 33%, *p* = 0.03 Fig. 4f).

A sensitivity analysis limited to RCT and prospective studies resulted in blood loss, transfusions, conversions, and readmissions becoming non-significant (Online Resource 4). Complications, operative time, mortality, and reoperation were still comparable, and length of hospital stay was about 7 h shorter in the robotic group (7 studies, 377 robotic, 465 laparoscopic patients, MD: – 0.28 [– 0.51, – 0.05], *I*^2^ = 86%, *p* = 0.02).

#### Robotic versus open approach

**Fig. 5:**
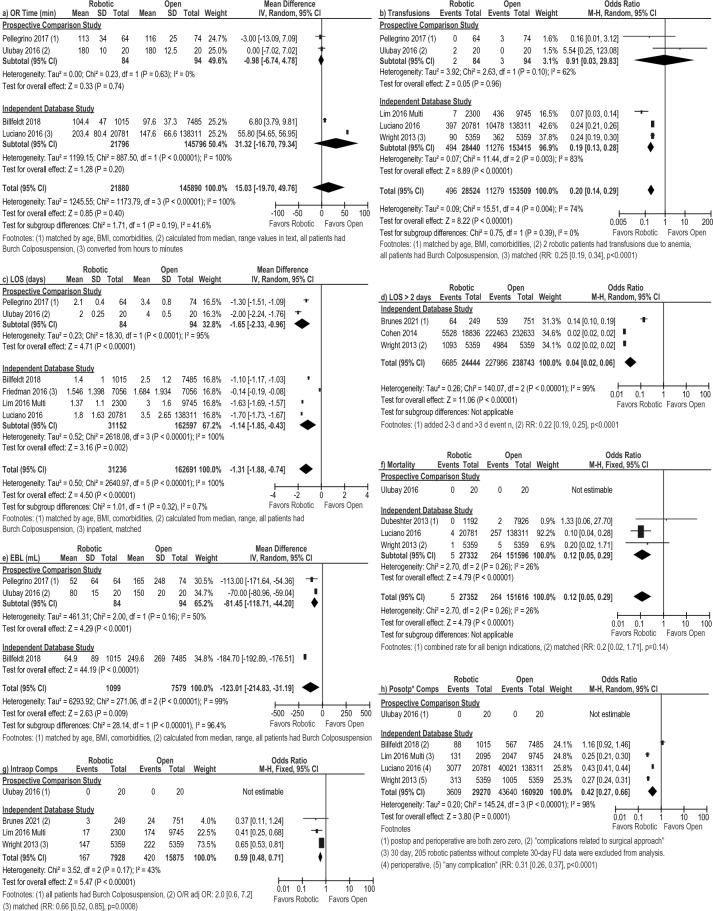
Forest plots for the robotic versus open surgery comparison showing the results of the overall pooled results and the study type subgroup analyses for **a** operative (OR) time in minutes (min), **b** blood transfusions, **c** length of hospital stay (LOS) in days, and **d** the number of patients requiring a hospital stay greater than 2 days, **e** estimated blood loss (EBL) in milliliters (mL), **f** mortality, **g** intraoperative complications (Intraop Comps), and **h** postoperative complications (Postop* Comps). *SD* standard deviation, *IV* inverse variance, *CI* confidence interval, *BMI* body mass index, *RR* risk ratio, *O* open, *R* robotic, *FU* follow-up. *Indicates that the postoperative complication rate within 30 days was extracted preferentially, but if the paper only reported a perioperative rate, it was included so as to not exclude that paper from the analysis

**Fig. 6:**
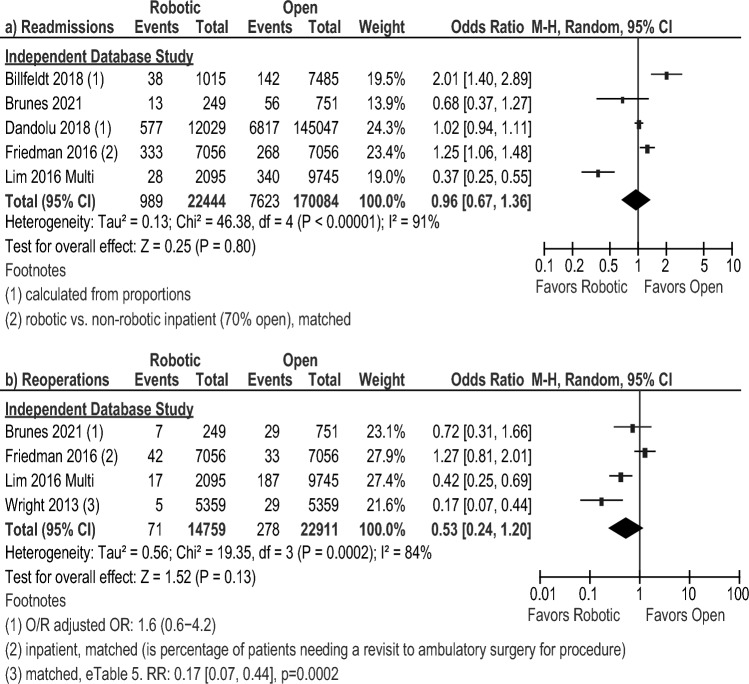
Forest plots for the robotic versus open surgery comparison showing the results of the overall pooled results and the study type subgroup analyses for **a** readmissions and **b** reoperations. *M–H* Mantel–Haenszel, *CI* confidence interval, *O* open, *R* robotic, *OR* odds ratio

Operative time was comparable in the overall pooled analysis and in all of the subgroup analyses (Fig. 5a). The overall pooled analysis and the database subgroup analysis for blood transfusions showed lower risk in the robotic group (Fig. 5b), but the subgroup analysis of prospective comparison studies was not significant. The individual meta-analyses of two prospective studies [34, 38] and four database studies [16, 24, 28, 30] reporter a shorter LOS favoring the robotic approach (MD: – 1.65 days [– 2.33, – 0.96], *I*^2^ = 95%, *p* < 0.00001 and MD: -1.14 days [– 1.85, – 0.43], *I*^2^ = 100%, *p* = 0.002, respectively**, **Fig. 5c) as did the overall pooled analysis (MD: – 1.31 [– 1.88, – 0.74], *I*^2^ = 100%, p < 0.00001). Consistent with this, the meta-analysis of three database studies [17, 19, 39] showed that the risk of staying more than 2 days in the hospital after the surgery was higher after an open approach (OR: 0.04 [0.02, 0.06], *I*^2^ = 99%, *p* < 0.00001, Fig. 5d). The meta-analyses of two prospective studies [34, 38] and the overall pooled analysis showed a lower EBL with the robotic approach versus the open approach (MD: – 81.45 mL [– 118.71, – 44.20], *I*^2^ = 50%, *p* < 0.0001, MD: – 123.01 [– 214.83, – 31.19], *I*^2^ = 99%, *p* = 0.009 respectively, Fig. 5e). The overall pooled analysis (three database studies [22, 30, 39] and one prospective study [38]) showed a lower risk of mortality with the robotic group (OR: 0.12 [0.05, 0.29], *I*^2^ = 26%, *p* < 0.00001, Fig. 5f). This difference persisted using a risk difference analysis (RD: – 0.0010 [– 0.0019, – 0.0002], *I*^2^ = 62%, *p* = 0.01). The risk of intraoperative and postoperative complications was found to be lower with the robotic approach based on database studies (OR: 0.59 [0.48, 0.71], *I*^2^ = 43%, *p* < 0.00001, Fig. 5g, and OR: 0.42 [0.27, 0.66], *I*^2^ = 98%, *p* = 0.0001, Fig. 5h, respectively). This difference persisted when using a risk difference analysis (intraoperative RD: – 0.01 [– 0.02, – 0.01], *I*^2^ = 0%, *p* < 0.00001; postoperative RD: – 0.09 [– 0.14, – 0.04], *I*^2^ = 98%, *p* = 0.0003). However, there was no difference in readmission or reoperation between the open and the robotic approach (Fig. 6).

#### Robotic versus vaginal approach

**Fig. 7:**
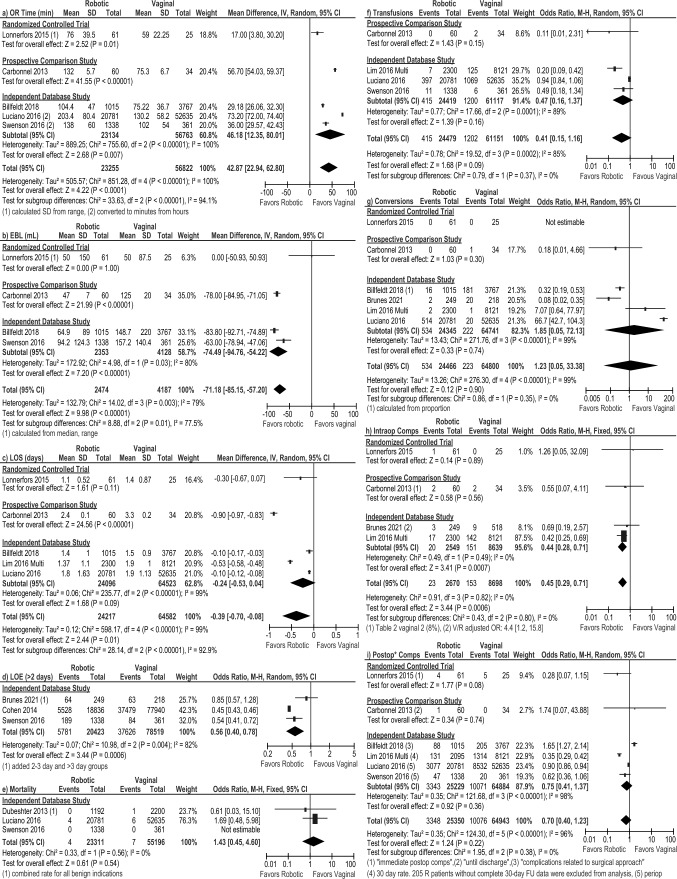
Forest plots for the robotic versus vaginal surgery comparison showing the results of the overall pooled results and the study type subgroup analyses for **a** operative (OR) time in minutes (min), **b** estimated blood loss (EBL) in milliliters (mL), **c** length of hospital stay (LOS) in days, **d** the number of patients requiring a hospital stay greater than 2 days, **e** mortality, **f** blood transfusions, **g** conversions to open surgery, **h** intraoperative complications (Intraop Comps), and **i** postoperative complications (Postop* Comps). *SD* standard deviation, *IV* inverse variance, *CI* confidence interval, *V* vaginal, *R* robotic, *OR* odds ratio, *FU* follow-up. *Indicates that the postoperative complication rate within 30 days was extracted preferentially, but if the paper only reported a perioperative rate (periop), it was included so as to not exclude that paper from the analysis

**Fig. 8:**
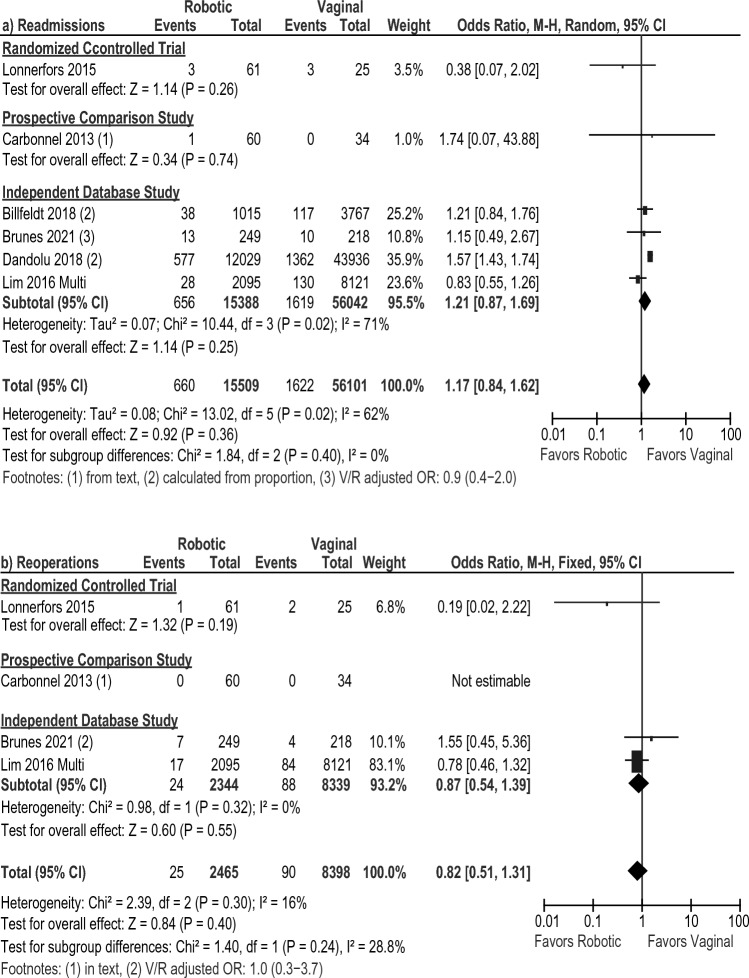
Forest plots for the robotic versus vaginal surgery comparison showing the results of the overall pooled results and the study type subgroup analyses for **a** readmissions and **b** reoperations. *M–H* Mantel–Haenszel, *CI* confidence interval, *V* vaginal, *R* robotic, *OR* odds ratio

Operative time was shown to be 17 min shorter with the vaginal approach compared to the robotic approach in 1 RCT [29] including 61 robotic and 25 vaginal patients (MD: 17 min [3.8, 30.2], *p* = 0.01, Fig. 7a). Similar results were found in one prospective study [18] and the meta-analysis of three database studies [16, 30, 37], for an overall pooled average savings of 43 min (MD: 42.87 [22.94, 62.80], *I*^2^ = 100%, *p* < 0.0001). EBL was lower with the robotic approach in the overall pooled result (MD: – 71.18 [– 85.15, – 57.20], *I*^2^ = 79%, *p* < 0.00001) and in the meta-analyses of two database studies [16, 37] (MD: – 74.49 mL [– 94.76, – 54.22], *I*^2^ = 80%, *p* < 0.00001, Fig. 7b). Similar results were found in one prospective study [18] (MD: – 78 mL [– 84.95, – 71.05], *p* < 0.00001) but one RCT [29] did not report any difference between the groups. Overall, LOS was shorter in the robotic group (MD: – 0.39 [– 0.70, – 0.08], *I*^2^ = 99%, *p* = 0.01, Fig. 7c), and in the one prospective study, but was comparable in the one RCT. The risk of prolonged LOS > 2 days was lower with the robotic approach (all database studies: OR: 0.56 [0.40, 0.78], *I*^2^ = 82%, *p* = 0.0006, Fig. 7d). There were no differences in mortality, blood transfusions, or conversions to the open approach (Fig. 7e–g). While intraoperative complications were comparable in one RCT [29] and one prospective study [18] (Fig. 7h), there was a lower risk of intraoperative complications during robotic procedures in the meta-analysis of two database studies [17, 28] (OR: 0.44 [0.28, 0.71], *I*^2^ = 0%, *p* = 0.0007) and in the overall pooled analysis (OR: 0.45 [0.29, 0.71], *I*^2^ = 0%, *p* = 0.0006), with no differences seen for postoperative complications (Fig. 7i). Overall, there were no significant differences in readmission or reoperation between the robotic and vaginal approaches (Fig. 8a, b).

Online Resource 5 shows the funnel plots for comparisons with at least ten studies. The funnel plots for the robotic vs. laparoscopic comparison for operative time and length of stay were inconclusive, but the funnel plots for the other parameters showed no publication bias.

## Discussion

In this systematic review and meta-analysis, we evaluated robotic-assisted benign hysterectomy versus laparoscopic, open, and vaginal surgery. The main strength of this systematic review is that it includes all randomized and prospective studies comparing the robotic approach to all the existing alternative surgical routes.

Our main findings were comparable outcomes between robotic and laparoscopic RCTs, shorter LOS, less EBL, and fewer complications compared to the open approach, and longer operative time but shorter LOS and less EBL compared to the vaginal approach.

Results of the meta-analysis comparing the robotic and the laparoscopic approach are consistent with the existing literature. As shown by Albright et al., when the analysis is limited to RCTs, the robotic approach was not found to significantly improve perioperative outcomes when compared to the laparoscopic approach [9]. Previous results of meta-analyses including mostly retrospective studies have been contradictory [6–8], and the meta-analysis of randomized studies settles this matter. Yet, one of the expected benefits of the robot was the reduction in operating time due to the improved surgical ergonomics enabled by the fully wristed instruments. However, the results of the included RCTs are contradictory and might be explained by the surgical learning curve. The two first RCTs [33, 36] published at the beginning of the robotic era reported longer operative time with the robotic platform, while Lonnerfors et al. reported shorter operative time with the robotic approach [29]. The authors state that they believe it was the experience of the surgeons and team that can explain this difference, which can result in a shorter setup, docking, and surgical intervention time. The democratization of the robot across different specialties and its increasingly widespread use should tend to reduce the operating time in the future.

Our meta-analysis comparing the robotic approach with the open approach reported the well-known benefits of any minimally invasive approach, with shorter LOS, less EBL, and fewer complications.

When vaginal or laparoscopic approaches are not feasible due to the complexity of the case, the use of the robotic platform might allow surgeons to avoid a laparotomy, and thus offer better postoperative outcomes to the patient. This is especially the case for selected patients with preoperative characteristics indicating potential surgical complexity, such as past surgical history, high BMI, or large uterus [40]. Although expert laparoscopic centers have reported the feasibility of this procedure for large uteruses [41, 42], the robotic approach is likely to overcome the drawbacks inherent to traditional laparoscopic surgery and its associated steep learning curve by providing magnified 3D vision, improved ergonomics, and wristed instruments. Thus, similar to what has been done in the setting of malignant uterine pathology [6], future studies should investigate the role of robotics by parsing out the results according to BMI, uterus weight, and other potential factors that contribute to surgical complexity.

In 2019, the ACOG (American Congress of Obstetricians and Gynecologists) reaffirmed 2017 and 2009 statements that the vaginal approach should be preferred for women undergoing hysterectomy for benign disease, "given its well-documented benefits and lower complication rates” [43]. While the AAGL (American Association of Gynecologic Laparoscopists) and the CNGOF (French National College of Obstetricians and Gynecologists) also advocate for the use of the vaginal or the laparoscopic approach as first-line choices in this setting [44]. However, there seems to be a discrepancy between the recommendations and the reality of the everyday practice. Indeed, use of the vaginal approach is in decline, demonstrating a consistent decrease in use from 25% of cases in 1998 to 17% of cases in 2010 [45]. In addition, a more recent nationwide analysis reported that vaginal cases decreased by 18.7% between 2016 and 2018 [1]. However, this surgical option remains important in the surgical arsenal, because it can be offered to patients unfit for laparotomy or laparoscopy due to comorbidity, or those unable to support prolonged Trendelenburg position or pneumoperitoneum.

Our meta-analysis is not devoid of limitations. First, meta-analyses comparing the robotic approach to the open and vaginal approach included mostly retrospective database studies. Pooling data from RCTs and prospective studies with retrospective studies is inherently problematic due to issues with bias with a retrospective study design, and in some cases, we saw high heterogeneity in the overall pooled analysis and differences in the subgroup analyses by study type. However, there were some outcomes where the specific test for subgroup differences was not significant, demonstrating consistency in the results for all study types. We also limited the retrospective studies that we included in the analysis to independent database studies that have safeguards in place to insure consistency, accuracy, and completeness in data entry [46]. We also provided a sensitivity analysis limited to RCT and prospective studies for the robotic versus laparoscopic comparison; however, there were not enough studies for the open and vaginal comparisons to perform this analysis, supporting the need for independent database studies. Second, the heterogeneity of the studies included in the meta-analysis was high and further exploratory statistical analyses were not performed. Last, this study did not include a cost-effectiveness analysis.

## Conclusion

Overall, while the robotic approach was mostly comparable to the laparoscopic route, it was associated with a shorter LOS, less EBL, and fewer complications when compared to the open approach. In comparison to the vaginal approach, the robotic route was associated with longer operative time but shorter LOS and less EBL. The robotic approach could be an opportunity to offer the perioperative benefits of minimally invasive surgery to patients who in the past would have been suitable only for an open approach, thus broadening the surgical armamentarium for benign hysterectomy.

### Supplementary Information

Below is the link to the electronic supplementary material.

Online Resource 1: Search methodology for systematic searches. Free-text protocol for combining robotic, indication, anatomic, and procedure terms to search for benign hysterectomy articles in Pubmed, Scopus, and Embase

Online Resource 2: Flowchart bibliography. A record of how references found in the search were designated in the flowchart for the 115 references assessed for eligibility

Online Resource 3: Newcastle Ottawa Score (NOS). A table with the risk of bias assessment results using the Newcastle Ottawa Score

Online Resource 4: Sensitivity analysis limited to RCT and prospective studies. A table with the number of studies and patients included in each analysis of outcomes with RCT or prospective studies, along with the effect size, 90% confidence interval, p-value, and heterogeneity analysis. Bolding indicates significance (p<0.05)

Online Resource 5: Funnel Plots. Funnel plots for each outcome with at least 10 individual studies included in the analysis

## Data Availability

All data are presented in the manuscript.

## References

[1] Wright JD, Huang Y, Li AH, Melamed A, Hershman DL (2022). Nationwide estimates of annual inpatient and outpatient hysterectomies performed in the United States. Obstet Gynecol.

[2] https://www.has-santefr/upload/docs/application/pdf/2021-12/rapport_hysterectomie_robot-assistee.pdf. Accessed 14 Jun 2023

[3] Cohen SL, Ajao MO, Clark NV, Vitonis AF, Einarsson JI (2017). Outpatient hysterectomy volume in the United States. Obstet Gynecol.

[4] Chevrot A, Margueritte F, Fritel X, Serfaty A, Huchon C, Fauconnier A (2021). Hysterectomy: practices evolution between 2009 and 2019 in France. Gynecol Obstet Fertil Senol.

[5] Gala RB, Margulies R, Steinberg A, Murphy M, Lukban J, Jeppson P, Aschkenazi S, Olivera C, South M, Lowenstein L, Schaffer J, Balk EM, Sung V, Society of Gynecologic Surgeons Systematic Review G (2014). Systematic review of robotic surgery in gynecology: robotic techniques compared with laparoscopy and laparotomy. J Minim Invasive Gynecol.

[6] O'Neill M, Moran PS, Teljeur C, O'Sullivan OE, O'Reilly BA, Hewitt M, Flattery M, Ryan M (2013). Robot-assisted hysterectomy compared to open and laparoscopic approaches: systematic review and meta-analysis. Arch Gynecol Obstet.

[7] Scandola M, Grespan L, Vicentini M, Fiorini P (2011). Robot-assisted laparoscopic hysterectomy vs traditional laparoscopic hysterectomy: five metaanalyses. J Minim Invasive Gynecol.

[8] Martino MA, Berger EA, McFetridge JT, Shubella J, Gosciniak G, Wejkszner T, Kainz GF, Patriarco J, Thomas MB, Boulay R (2014). A comparison of quality outcome measures in patients having a hysterectomy for benign disease: robotic vs. non-robotic approaches. J Minim Invasive Gynecol.

[9] Albright BB, Witte T, Tofte AN, Chou J, Black JD, Desai VB, Erekson EA (2016). Robotic versus laparoscopic hysterectomy for benign disease: a systematic review and meta-analysis of randomized trials. J Minim Invasive Gynecol.

[10] Liu H, Lawrie TA, Lu D, Song H, Wang L, Shi G (2014) Robot-assisted surgery in gynaecology. Cochrane Database Syst Rev (12):CD011422 10.1002/14651858.CD011422 PMC6457792 25493418

[11] Tapper AM, Hannola M, Zeitlin R, Isojarvi J, Sintonen H, Ikonen TS (2014). A systematic review and cost analysis of robot-assisted hysterectomy in malignant and benign conditions. Eur J Obstet Gynecol Reprod Biol.

[12] Moher D, Liberati A, Tetzlaff J, Altman DG, Group P (2009). Preferred reporting items for systematic reviews and meta-analyses: the PRISMA statement. Ann Intern Med.

[13] Payne TN, Dauterive FR, Pitter MC, Giep HN, Giep BN, Grogg TW, Shanbour KA, Goff DW, Hubert HB (2010). Robotically assisted hysterectomy in patients with large uteri: outcomes in five community practices. Obstet Gynecol.

[14] Higgins JP, Altman DG, Gotzsche PC, Juni P, Moher D, Oxman AD, Savovic J, Schulz KF, Weeks L, Sterne JA, Cochrane Bias Methods G, Cochrane Statistical Methods G (2011). The Cochrane Collaboration's tool for assessing risk of bias in randomised trials. BMJ.

[15] Wells GABS, D O'Connell, J Peterson, V Welch, M Losos, P Tugwell The Newcastle-Ottawa Scale (NOS) for assessing the quality of nonrandomised studies in meta-analyses. https://www.ohrica//programs/clinical_epidemiology/oxfordasp. Accessed 14 Jun 2023

[16] Billfeldt NK, Borgfeldt C, Lindkvist H, Stjerndahl JH, Ankardal M (2018). A Swedish population-based evaluation of benign hysterectomy, comparing minimally invasive and abdominal surgery. Eur J Obstet Gynecol Reprod Biol.

[17] Brunes M, Johannesson U, Habel H, Soderberg MW, Ek M (2021). Effects of obesity on peri- and postoperative outcomes in patients undergoing robotic vs. conventional hysterectomy. J Minim Invasive Gynecol.

[18] Carbonnel M, Abbou H, N'Guyen HT, Roy S, Hamdi G, Jnifen A, Ayoubi JM (2013). Robotically assisted hysterectomy versus vaginal hysterectomy for benign disease: a prospective study. Minim Invasive Surg.

[19] Cohen SL, Vitonis AF, Einarsson JI (2014) Updated hysterectomy surveillance and factors associated with minimally invasive hysterectomy. JSLS 18(3):e2014.00096 10.4293/JSLS.2014.00096 PMC4208898 25392662

[20] Dandolu V, Pathak P (2018). Health resource utilization and costs during the first 90 days following robot-assisted hysterectomy. Int Urogynecol J.

[21] Deimling TA, Eldridge JL, Riley KA, Kunselman AR, Harkins GJ (2017). Randomized controlled trial comparing operative times between standard and robot-assisted laparoscopic hysterectomy. Int J Gynaecol Obstet.

[22] Dubeshter B, Angel C, Toy E, Thomas S, Glantz JC (2013). Current role of robotic hysterectomy. J Gynecol Surg.

[23] Elessawy M, Schneekloth S, Günther V, Maass N, Mettler L, Alkatout I (2020). Postoperative telephone-based questionnaire on quality of life after robotic-assisted laparoscopic hysterectomy versus conventional total laparoscopic hysterectomy. J Clin Med.

[24] Friedman B, Barbash GI, Glied SA, Steiner CA (2016). Hospital revisits within 30 days after conventional and robotically assisted hysterectomy. Med Care.

[25] Hart S, Hashemi L, Sobolewski CJ (2013). Effect of a disposable automated suturing device on cost and operating room time in benign total laparoscopic hysterectomy procedures. Jsls.

[26] Herrinton LJ, Raine-Bennett T, Liu L, Alexeeff SE, Ramos W, Suh-Burgmann B (2020) Outcomes of robotic hysterectomy for treatment of benign conditions: influence of patient complexity. Perm J 24:19.035 10.7812/TPP/19.035 PMC6972554 31905335

[27] Lim CS, Mowers EL, Mahnert N, Skinner BD, Kamdar N, Morgan DM, As-Sanie S (2016). Risk factors and outcomes for conversion to laparotomy of laparoscopic hysterectomy in benign gynecology. Obstet Gynecol.

[28] Lim PC, Crane JT, English EJ, Farnam RW, Garza DM, Winter ML, Rozeboom JL (2016). Multicenter analysis comparing robotic, open, laparoscopic, and vaginal hysterectomies performed by high-volume surgeons for benign indications. Int J Gynaecol Obstet.

[29] Lonnerfors C, Reynisson P, Persson J (2015). A randomized trial comparing vaginal and laparoscopic hysterectomy vs robot-assisted hysterectomy. J Minim Invasive Gynecol.

[30] Luciano AA, Luciano DE, Gabbert J, Seshadri-Kreaden U (2016). The impact of robotics on the mode of benign hysterectomy and clinical outcomes. Int J Med Robot.

[31] Martinez-Maestre MA, Gambadauro P, Gonzalez-Cejudo C, Torrejon R (2014). Total laparoscopic hysterectomy with and without robotic assistance: a prospective controlled study. Surg Innov.

[32] Ngan TYT, Zakhari A, Czuzoj-Shulman N, Tulandi T, Abenhaim HA (2018). Laparoscopic and robotic-assisted hysterectomy for uterine leiomyomas: a comparison of complications and costs. J Obstet Gynaecol Can.

[33] Paraiso MF, Ridgeway B, Park AJ, Jelovsek JE, Barber MD, Falcone T, Einarsson JI (2013). A randomized trial comparing conventional and robotically assisted total laparoscopic hysterectomy. Am J Obstet Gynecol.

[34] Pellegrino A, Damiani GR, Fachechi G, Corso S, Pirovano C, Trio C, Villa M, Turoli D, Youssef A (2017). Cost analysis of minimally invasive hysterectomy vs open approach performed by a single surgeon in an Italian center. J Robot Surg.

[35] Rosero EB, Kho KA, Joshi GP, Giesecke M, Schaffer JI (2013). Comparison of robotic and laparoscopic hysterectomy for benign gynecologic disease. Obstet Gynecol.

[36] Sarlos D, Kots L, Stevanovic N, von Felten S, Schar G (2012). Robotic compared with conventional laparoscopic hysterectomy: a randomized controlled trial. Obstet Gynecol.

[37] Swenson CW, Kamdar NS, Harris JA, Uppal S, Campbell DA, Morgan DM (2016). Comparison of robotic and other minimally invasive routes of hysterectomy for benign indications. Am J Obstet Gynecol.

[38] Ulubay M, Dede M, Ozturk M, Keskin U, Fidan U, Alanbay I, Yenen MC (2016). Comparison of robotic-assisted and abdominal hysterectomy with concomitant burch colposuspension. J Gynecol Surg.

[39] Wright JD, Ananth CV, Lewin SN, Burke WM, Lu YS, Neugut AI, Herzog TJ, Hershman DL (2013). Robotically assisted vs laparoscopic hysterectomy among women with benign gynecologic disease. JAMA.

[40] Driessen SR, Sandberg EM, la Chapelle CF, Twijnstra AR, Rhemrev JP, Jansen FW (2016). Case-mix variables and predictors for outcomes of laparoscopic hysterectomy: a systematic review. J Minim Invasive Gynecol.

[41] Lambat Emery S, Boulvain M, Petignat P, Dubuisson J (2021). Operative complications and outcomes comparing small and large uterine weight in case of laparoscopic hysterectomy for a benign indication. Front Surg.

[42] Louie M, Strassle PD, Moulder JK, Dizon AM, Schiff LD, Carey ET (2018). Uterine weight and complications after abdominal, laparoscopic, and vaginal hysterectomy. Am J Obstet Gynecol.

[43] (2017) Committee opinion: choosing the Route of Hysterectomy for Benign Disease. https://www.acogorg/clinical/clinical-guidance/committee-opinion/articles/2017/06/choosing-the-route-of-hysterectomy-for-benign-disease. Accessed 14 Jun 2023

[44] Deffieux X, Rochambeau B, Chene G, Gauthier T, Huet S, Lamblin G, Agostini A, Marcelli M, Golfier F (2016). Hysterectomy for benign disease: clinical practice guidelines from the French College of Obstetrics and Gynecology. Eur J Obstet Gynecol Reprod Biol.

[45] Wright JD, Herzog TJ, Tsui J, Ananth CV, Lewin SN, Lu YS, Neugut AI, Hershman DL (2013). Nationwide trends in the performance of inpatient hysterectomy in the United States. Obstet Gynecol.

[46] Real-World Data: Assessing Registries to Support Regulatory Decision-Making for Drug and Biological Products: Guidance for Industry” U.S. Department of Health and Human Services FDA. (2021) Available at: Real-World Data: Assessing Registries to Support Regulatory Decision-Making for Drug and Biological Products Guidance for Industry | FDA. Accessed 5 June 2022

