# Supplementary material for: Robotic-assisted benign hysterectomy compared with laparoscopic, vaginal, and open surgery: a systematic review and meta-analysis
Source: J Robot Surg. 2023 Oct 19;17(6):2647–62. doi: 10.1007/s11701-023-01724-6 (PMC10678826; doi:10.1007/s11701-023-01724-6)
Supplement: Supplementary file 1 — Online Resource 1: Search methodology for systematic searches. Free-text protocol for combining robotic, indication, anatomic, and procedure terms to search for benign hysterectomy articles in Pubmed, Scopus, and Embase [file 11701_2023_1724_MOESM1_ESM.docx]

Robotic-assisted benign hysterectomy compared with laparoscopic, vaginal, and open surgery: A systematic review and meta-analysis. Journal of Robotic Surgery

Louis Lenfant^1,2^, Geoffroy Canlorbe^2^, Jérémie Belghiti^2^, Usha Seshadri Kreaden^3^, April E. Hebert^3^, Marianne Nikpayam^2^, Catherine Uzan^2^, Henri Azaïs^2,4*^

1 Sorbonne Université, Department of Urology, Academic Hospital Pitié-Salpêtrière, APHP, F-75013 PARIS, France

2 Department of Surgery and Oncological Gynecology, Pitié-Salpétrière University Hospital, Assistance Publique des Hôpitaux de Paris, Sorbonne University, Paris, France

3 Biostatistics & Global Evidence Management, Intuitive Surgical Inc, Sunnyvale, California

4 Gynecologic and Breast Oncologic Surgery Department, Georges Pompidou European Hospital, APHP. Centre, Université de Paris Cité, Paris, France

*Corresponding author E-mail: henriazais@gmail.com (HA)

Benign Hysterectomy Pubmed Search (1/1/2010-12/31/2020)

| **Search Steps** | **Pubmed Search Terms (in Advanced search, all fields search is default)** | **Search Results 06/02/2021** |
| --- | --- | --- |
| #1 | In All Fields: (Robot* OR "da vinci" OR davinci OR "Intuitive Surgical") | 71,382 |
| #2 | In Title/Abstract: (endometrial OR endometrium OR endometrioid OR uterus OR uterine OR gynecology OR gynecological OR gynecologic OR gynaecology OR gynaecologic OR gynaecological OR adnexal) | 302,132 |
| #3 | In All Fields: (benign OR prolapse* OR myom* OR leiomyoma* OR fibroid OR fibroids OR endometriosis OR amenorrhea OR dysmenorrhea OR polycyst* OR “pelvic inflammatory disease” OR masses OR mass OR tumor OR tumour OR sacro* OR sacra* OR adhesions OR "pelvic pain" OR "postmenopausal bleeding" OR "post-menopausal bleeding" OR "post menopausal bleeding") | 6,041,095 |
| #4 | Title/Abstract: (surgery OR surgical OR surgeries) | 1,959,320 |
| #5 | #3 OR #4 | 7,363,482 |
| #6 | (Title/Abstract: (cancer OR carcinoma OR adenocarcinoma OR cancerous OR malignant OR malignancy OR neoplasm OR neoplasia OR oncology OR oncologic OR oncological OR oncol)) NOT (Title/Abstract: (benign OR prolapse* OR myom* OR leiomyoma* OR fibroid OR fibroids OR endometriosis OR amenorrhea OR dysmenorrhea OR polycyst* OR ‘pelvic inflammatory disease’ OR masses OR mass OR tumor OR tumour OR sacro* OR sacra* OR adhesions OR "pelvic pain" OR "postmenopausal bleeding" OR "post-menopausal bleeding" OR "post menopausal bleeding")) | 1,681,045 |
| #7 | #1 AND #2 AND #5 | 2,057 |
| #8 | #7 NOT #6 | 1,470 |
| #9 | In Title/Abstract: Hysterectomy OR hysterectomies | 38,492 |
| #10 | (Title: (cancer OR carcinoma OR adenocarcinoma OR cancerous OR malignant OR malignancy OR neoplasm OR neoplasia OR oncology OR oncologic OR oncological OR oncol)) NOT (Title/Abstract: (benign OR prolapse* OR myom* OR leiomyoma* OR fibroid OR fibroids OR endometriosis OR amenorrhea OR dysmenorrhea OR polycyst* OR ‘pelvic inflammatory disease’ OR masses OR mass OR tumor OR tumour OR sacro* OR sacra* OR adhesions OR "pelvic pain" OR "postmenopausal bleeding" OR "post-menopausal bleeding" OR "post menopausal bleeding")) | 1,054,474 |
| #11 | #1 AND #9 | 1,426 |
| #12 | #11 NOT #10 | 1,143 |
| #13 | #8 OR #12 | 1,994 |
| #14 | #13 AND ("2010/01/01"[PDat] : "2020/12/31"[PDat]) | 1,697 |
| #15 | #14 AND English[lang] | 1,635 |
|  | **Pubmed Search (quality control step)** |  |
| QC | (((((Robot* OR "da vinci" OR davinci OR "Intuitive Surgical") AND (endometrial[Title/Abstract] OR endometrium[Title/Abstract] OR endometrioid[Title/Abstract] OR uterus[Title/Abstract] OR uterine[Title/Abstract] OR gynecology[Title/Abstract] OR gynecological[Title/Abstract] OR gynecologic[Title/Abstract] OR gynaecology[Title/Abstract] OR gynaecologic[Title/Abstract] OR gynaecological[Title/Abstract] OR adnexal[Title/Abstract])) AND ((benign OR prolapse* OR myom* OR leiomyoma* OR fibroid OR fibroids OR endometriosis OR amenorrhea OR dysmenorrhea OR polycyst* OR "pelvic inflammatory disease" OR masses OR mass OR tumor OR tumour OR sacro* OR sacra* OR adhesions OR "pelvic pain" OR "postmenopausal bleeding" OR "post-menopausal bleeding" OR "post menopausal bleeding") OR (surgery[Title/Abstract] OR surgical[Title/Abstract] OR surgeries[Title/Abstract]))) NOT ((cancer[Title/Abstract] OR carcinoma[Title/Abstract] OR adenocarcinoma[Title/Abstract] OR cancerous[Title/Abstract] OR malignant[Title/Abstract] OR malignancy[Title/Abstract] OR neoplasm[Title/Abstract] OR neoplasia[Title/Abstract] OR oncology[Title/Abstract] OR oncologic[Title/Abstract] OR oncological[Title/Abstract] OR oncol[Title/Abstract]) NOT (benign[Title/Abstract] OR prolapse*[Title/Abstract] OR myom*[Title/Abstract] OR leiomyoma*[Title/Abstract] OR fibroid[Title/Abstract] OR fibroids[Title/Abstract] OR endometriosis[Title/Abstract] OR amenorrhea[Title/Abstract] OR dysmenorrhea[Title/Abstract] OR polycyst*[Title/Abstract] OR ‘pelvic inflammatory disease’[Title/Abstract] OR masses[Title/Abstract] OR mass[Title/Abstract] OR tumor[Title/Abstract] OR tumour[Title/Abstract] OR sacro*[Title/Abstract] OR sacra*[Title/Abstract] OR adhesions[Title/Abstract] OR "pelvic pain"[Title/Abstract] OR "postmenopausal bleeding"[Title/Abstract] OR "post-menopausal bleeding"[Title/Abstract] OR "post menopausal bleeding"[Title/Abstract]))) OR (((Robot* OR "da vinci" OR davinci OR "Intuitive Surgical") AND (Hysterectomy[Title/Abstract] OR hysterectomies[Title/Abstract])) NOT ((cancer[Title] OR carcinoma[Title] OR adenocarcinoma[Title] OR cancerous[Title] OR malignant[Title] OR malignancy[Title] OR neoplasm[Title] OR neoplasia[Title] OR oncology[Title] OR oncologic[Title] OR oncological[Title] OR oncol[Title]) NOT (benign[Title/Abstract] OR prolapse*[Title/Abstract] OR myom*[Title/Abstract] OR leiomyoma*[Title/Abstract] OR fibroid[Title/Abstract] OR fibroids[Title/Abstract] OR endometriosis[Title/Abstract] OR amenorrhea[Title/Abstract] OR dysmenorrhea[Title/Abstract] OR polycyst*[Title/Abstract] OR ‘pelvic inflammatory disease’[Title/Abstract] OR masses[Title/Abstract] OR mass[Title/Abstract] OR tumor[Title/Abstract] OR tumour[Title/Abstract] OR sacro*[Title/Abstract] OR sacra*[Title/Abstract] OR adhesions[Title/Abstract] OR "pelvic pain"[Title/Abstract] OR "postmenopausal bleeding"[Title/Abstract] OR "post-menopausal bleeding"[Title/Abstract] OR "post menopausal bleeding"[Title/Abstract]))) AND ("2010/01/01"[PDat] : "2020/12/31"[PDat])) AND (English[Language]) | 1,635 |

Benign Hysterectomy Scopus Search (1/1/2010-12/31/2020)

| **Search Steps** | **Scopus Search Terms (copy into advance search box)** | **Search Results 06/02/2021** |
| --- | --- | --- |
| #1 | TITLE-ABS-KEY (da*vinci) OR (robotic AND surgery) OR ("intuitive surgical") OR (robotic AND assist*) OR (robot*surgery) OR (robotic-assist*) | 225,994 |
| #2 | TITLE-ABS-KEY ( ( da*vinci ) OR ( intuitive W/3 surgical ) OR ( robot* W/2 assist* ) OR ( robot*assist* ) OR ( robot* W/8 ( surgery OR surgeries OR surgical* OR bypass OR graft OR sentinel OR *ectomy OR *otomy OR *plasty OR *opexy OR cancer OR tumor OR neoplasm* OR excis* OR anastomosis OR benign OR malignanc* ) ) OR endowrist OR 'dv-trainer' ) OR MANUFACTURER ( intuitive ) OR TRADENAME ( 'da AND vinci' OR davinci ) OR AFFIL ( intuitive OR intusurg ) OR REFTITLE ( ( da*vinci ) OR ( intuitive W/3 surgical ) OR ( robot*assist* ) OR ( ( robot* ) W/8 ( surgery OR surgeries OR surgical OR cancer OR bypass OR graft OR sentinel OR *ectomy OR *otomy OR *plasty OR *opexy OR tumor OR cancer OR neoplasm* ) ) OR endowrist OR 'dv-trainer' ) OR ( EXACTSRCTITLE ( 'health AND technology AND assessment' ) AND TITLE-ABS-KEY ( surgery OR surgeries OR surgical ) ) | 96,709 |
| #3 | TITLE-ABS-KEY ( endometrial OR endometrium OR endometrioid OR uterus OR uterine OR gynecology OR gynecological OR gynecologic OR gynaecology OR gynaecological OR gynaecologic OR adnexal OR hysterectom* ) AND ALL (benign OR masses OR tumor OR tumour OR sacra* OR sacro* OR prolapse* OR myom* OR leiomyoma* OR fibroid OR fibroids OR endometriosis OR amenorrhea OR dysmenorrhea OR polycyst* OR “pelvic inflammatory disease” OR adhesions OR "pelvic pain" OR "postmenopausal bleeding" OR "post-menopausal bleeding" OR "post menopausal bleeding") | 330,978 |
| #4 | ( REF ( ( robot OR robotic OR robotically OR "da vinci" OR "intuitive surgical" ) W/3 ( endometrial OR endometrium OR endometrioid OR uterine OR uterus OR gynecology OR gynecological OR gynecologic OR gynaecological OR gynaecologic OR gynaecology OR adnexal OR hysterectomy OR hysterectomies) ) ) AND ( REF ( ( robot OR robotic OR robotically OR "da vinci" OR "intuitive surgical" ) W/3 (benign OR masses OR tumor OR tumour OR sacra* OR sacro* OR prolapse* OR myom* OR leiomyoma* OR fibroid OR fibroids OR endometriosis OR amenorrhea OR dysmenorrhea OR polycyst* OR “pelvic inflammatory disease” OR adhesions OR "pelvic pain" OR "postmenopausal bleeding" OR "post-menopausal bleeding" OR "post menopausal bleeding") ) ) | 1,139 |
| #5 | TITLE-ABS-KEY ( ( robot OR robotic OR robotically OR "da vinci" OR "intuitive surgical" ) AND ( hysterectomy OR hysterectomies )) | 2,362 |
| #6 | TITLE ( cancer OR carcinoma OR adenocarcinoma OR cancerous OR malignant OR malignancy OR neoplasm OR neoplasia OR oncology OR oncologic OR oncological OR oncol ) AND NOT TITLE-ABS ( benign OR prolapse* OR myom* OR leiomyoma* OR fibroid OR fibroids OR endometriosis OR amenorrhea OR dysmenorrhea OR polycyst* OR "pelvic inflammatory disease" OR sacro* OR sacra* OR adhesions OR "pelvic pain") | 1,925,915 |
| #7 | ((#1 OR #2) AND (#3 OR #4)) OR #5 | 7,107 |
| #8 | #7 AND PUBYEAR > 2009 AND PUBYEAR < 2021 | 6,225 |
| #9 | #8 AND NOT #6 | 4,358 |
| #10 | #9 Exclude books, book series, book chapters and multivolume references using filters for document type and source type | 4,150 |
| #11 | #10 Limit to English using filters | 3,926 |
|  | Scopus Search (quality control step) (copy into advance search box) |  |
| QC | ( ( ( ( TITLE-ABS-KEY ( da*vinci ) OR ( robotic AND surgery ) OR ( "intuitive surgical" ) OR ( robotic AND assist* ) OR ( robot*surgery ) OR ( robotic-assist* ) ) OR ( TITLE-ABS-KEY ( ( da*vinci ) OR ( intuitive W/3 surgical ) OR ( robot* W/2 assist* ) OR ( robot*assist* ) OR ( robot* W/8 ( surgery OR surgeries OR surgical* OR bypass OR graft OR sentinel OR *ectomy OR *otomy OR *plasty OR *opexy OR cancer OR tumor OR neoplasm* OR excis* OR anastomosis OR benign OR malignanc* ) ) OR endowrist OR 'dv-trainer' ) OR MANUFACTURER ( intuitive ) OR TRADENAME ( 'da AND vinci' OR davinci ) OR AFFIL ( intuitive OR intusurg ) OR REFTITLE ( ( da*vinci ) OR ( intuitive W/3 surgical ) OR ( robot*assist* ) OR ( ( robot* ) W/8 ( surgery OR surgeries OR surgical OR cancer OR bypass OR graft OR sentinel OR *ectomy OR *otomy OR *plasty OR *opexy OR tumor OR cancer OR neoplasm* ) ) OR endowrist OR 'dv-trainer' ) OR ( EXACTSRCTITLE ( 'health AND technology AND assessment' ) AND TITLE-ABS-KEY ( surgery OR surgeries OR surgical ) ) ) ) AND ( ( TITLE-ABS-KEY ( endometrial OR endometrium OR endometrioid OR uterus OR uterine OR gynecology OR gynecological OR gynecologic OR gynaecology OR gynaecological OR gynaecologic OR adnexal OR hysterectom* ) AND ALL ( benign OR masses OR tumor OR tumour OR sacra* OR sacro* OR prolapse* OR myom* OR leiomyoma* OR fibroid OR fibroids OR endometriosis OR amenorrhea OR dysmenorrhea OR polycyst* OR "pelvic inflammatory disease" OR adhesions OR "pelvic pain" OR "postmenopausal bleeding" OR "post-menopausal bleeding" OR "post menopausal bleeding" ) ) OR ( ( REF ( ( robot OR robotic OR robotically OR "da vinci" OR "intuitive surgical" ) W/3 ( endometrial OR endometrium OR endometrioid OR uterine OR uterus OR gynecology OR gynecological OR gynecologic OR gynaecological OR gynaecologic OR gynaecology OR adnexal OR hysterectomy OR hysterectomies ) ) ) AND ( REF ( ( robot OR robotic OR robotically OR "da vinci" OR "intuitive surgical" ) W/3 ( benign OR masses OR tumor OR tumour OR sacra* OR sacro* OR prolapse* OR myom* OR leiomyoma* OR fibroid OR fibroids OR endometriosis OR amenorrhea OR dysmenorrhea OR polycyst* OR "pelvic inflammatory disease" OR adhesions OR "pelvic pain" OR "postmenopausal bleeding" OR "post-menopausal bleeding" OR "post menopausal bleeding" ) ) ) ) ) ) OR ( TITLE-ABS-KEY ( ( robot OR robotic OR robotically OR "da vinci" OR "intuitive surgical" ) AND ( hysterectomy OR hysterectomies ) ) ) AND PUBYEAR > 2009 AND PUBYEAR < 2021 ) AND NOT ( TITLE ( cancer OR carcinoma OR adenocarcinoma OR cancerous OR malignant OR malignancy OR neoplasm OR neoplasia OR oncology OR oncologic OR oncological OR oncol ) AND NOT TITLE-ABS ( benign OR prolapse* OR myom* OR leiomyoma* OR fibroid OR fibroids OR endometriosis OR amenorrhea OR dysmenorrhea OR polycyst* OR "pelvic inflammatory disease" OR sacro* OR sacra* OR adhesions OR "pelvic pain" ) ) AND ( EXCLUDE ( DOCTYPE , "ch" ) OR EXCLUDE ( DOCTYPE , "bk" ) ) AND ( EXCLUDE ( SRCTYPE , "b" ) OR EXCLUDE ( SRCTYPE , "k" ) ) AND ( LIMIT-TO ( LANGUAGE , "English" ) ) | 3,926 |

Benign Hysterectomy Embase Search (1/1/2010-12/31/2020)

|  | **Embase Search Terms (copy into advance search box)** | **Search Results**  **06/02/2021** |
| --- | --- | --- |
| #1 | ('da vinci':de,nc,lnk,cl,ab,ti OR 'davinci':de,nc,lnk,cl,ab,ti OR 'intuitive surgical' OR 'endowrist'/exp OR endowrist OR ((robot* NEAR/2 surg*):de,nc,lnk,cl,ab,ti) OR (robot* NEXT/1 assist*)) NOT (arthropl* OR 'gait'/exp OR gait) | 46,428 |
| #2 | robot OR robotic OR robotically | 79,561 |
| #3 | #1 OR #2 | 80,906 |
| #4 | (endometrial OR endometrium OR endometrioid OR uterus OR uterine OR gynecolo* OR gynaecolo* OR adnexal OR hysterectom*):ti,ab,kw | 458,147 |
| #5 | benign OR prolapse* OR myom* OR leiomyoma* OR fibroid OR fibroids OR endometriosis OR amenorrhea OR dysmenorrhea OR polycyst* OR “pelvic inflammatory disease” OR masses OR mass OR tumor OR tumour OR sacro* OR sacra* OR adhesions OR "pelvic pain" OR "postmenopausal bleeding" OR "post-menopausal bleeding" OR "post menopausal bleeding" | 5,506,360 |
| #6 | hysterectomy OR hysterectomies | 89,311 |
| #7 | (hysterectomy OR hysterectomies):ti,ab,kw | 60,672 |
| #8 | (#3 AND #4 AND #5) OR (#3 AND #5 AND #6) OR (#3 AND #7) | 5,458 |
| #9 | #8 AND [2010-2020]/py | 5,045 |
| #10 | #9 AND [english]/lim | 4,976 |
| #11 | #10 NOT [conference abstract]/lim | 2,038 |
|  | **Embase Search (quality control step) (copy into advance search box)** |  |
| QC | ((('da vinci':de,nc,lnk,cl,ab,ti OR 'davinci':de,nc,lnk,cl,ab,ti OR 'intuitive surgical' OR 'endowrist' OR 'endowrist'/exp OR endowrist OR ((robot* NEAR/2 surg*):de,nc,lnk,cl,ab,ti) OR (robot* NEXT/1 assist*)) NOT (arthropl* OR 'gait' OR 'gait'/exp OR gait) OR robot OR robotic OR robotically) AND (endometrial:ti,ab,kw OR endometrium:ti,ab,kw OR endometrioid:ti,ab,kw OR uterus:ti,ab,kw OR uterine:ti,ab,kw OR gynecolo*:ti,ab,kw OR gynaecolo*:ti,ab,kw OR adnexal:ti,ab,kw OR hysterectom*:ti,ab,kw) AND (benign OR prolapse* OR myom* OR leiomyoma* OR fibroid OR fibroids OR endometriosis OR amenorrhea OR dysmenorrhea OR polycyst* OR 'pelvic inflammatory disease' OR masses OR mass OR tumor OR tumour OR sacro* OR sacra* OR adhesions OR 'pelvic pain' OR 'postmenopausal bleeding' OR 'post-menopausal bleeding' OR 'post menopausal bleeding') OR ((('da vinci':de,nc,lnk,cl,ab,ti OR 'davinci':de,nc,lnk,cl,ab,ti OR 'intuitive surgical' OR 'endowrist' OR 'endowrist'/exp OR endowrist OR ((robot* NEAR/2 surg*):de,nc,lnk,cl,ab,ti) OR (robot* NEXT/1 assist*)) NOT (arthropl* OR 'gait' OR 'gait'/exp OR gait) OR robot OR robotic OR robotically) AND (benign OR prolapse* OR myom* OR leiomyoma* OR fibroid OR fibroids OR endometriosis OR amenorrhea OR dysmenorrhea OR polycyst* OR 'pelvic inflammatory disease' OR masses OR mass OR tumor OR tumour OR sacro* OR sacra* OR adhesions OR 'pelvic pain' OR 'postmenopausal bleeding' OR 'post-menopausal bleeding' OR 'post menopausal bleeding') AND (hysterectomy OR hysterectomies)) OR ((('da vinci':de,nc,lnk,cl,ab,ti OR 'davinci':de,nc,lnk,cl,ab,ti OR 'intuitive surgical' OR 'endowrist' OR 'endowrist'/exp OR endowrist OR ((robot* NEAR/2 surg*):de,nc,lnk,cl,ab,ti) OR (robot* NEXT/1 assist*)) NOT (arthropl* OR 'gait' OR 'gait'/exp OR gait) OR robot OR robotic OR robotically) AND (hysterectomy:ti,ab,kw OR hysterectomies:ti,ab,kw))) AND [2010-2020]/py AND [english]/lim NOT [conference abstract]/lim | 2,038 |
