# Supplementary material for: Robotic-assisted benign hysterectomy compared with laparoscopic, vaginal, and open surgery: a systematic review and meta-analysis
Source: J Robot Surg. 2023 Oct 19;17(6):2647–62. doi: 10.1007/s11701-023-01724-6 (PMC10678826; doi:10.1007/s11701-023-01724-6)
Supplement: Supplementary file 2 — Online Resource 2: Flowchart bibliography. A record of how references found in the search were designated in the flowchart for the 115 references assessed for eligibility [file 11701_2023_1724_MOESM2_ESM.docx]

Robotic-assisted benign hysterectomy compared with laparoscopic, vaginal, and open surgery: A systematic review and meta-analysis. Journal of Robotic Surgery.

Louis Lenfant1,2, Geoffroy Canlorbe2, Jérémie Belghiti2, Usha Seshadri Kreaden3, April E. Hebert3, Marianne Nikpayam2, Catherine Uzan2, Henri Azaïs2,4*

1 Sorbonne Université, Department of Urology, Academic Hospital Pitié-Salpêtrière, APHP, F-75013 PARIS, France

2 Department of Surgery and Oncological Gynecology, Pitié-Salpétrière University Hospital, Assistance Publique des Hôpitaux de Paris, Sorbonne University, Paris, France

3 Biostatistics & Global Evidence Management, Intuitive Surgical Inc, Sunnyvale, California

4 Gynecologic and Breast Oncologic Surgery Department, Georges Pompidou European Hospital, APHP. Centre, Université de Paris Cité, Paris, France

*Corresponding author E-mail: henriazais@gmail.com (HA)

**Eligibility Flowchart, n=115**

| **#** | **Flowchart Designation** | **Citation** |
| --- | --- | --- |
| 1 | Excluded - No lap, open, or vaginal comparison cohort | Alemzadeh, H., J. Raman, N. Leveson, Z. Kalbarczyk and R. K. Iyer (2016). "Adverse Events in Robotic Surgery: A Retrospective Study of 14 Years of FDA Data." PLoS One 11(4): e0151470. |
| 2 | Excluded - No lap, open, or vaginal comparison cohort | Fieber, J. H., L. E. Kuo, C. Wirtalla and R. R. Kelz (2020). "Variation in the utilization of robotic surgical operations." Journal of robotic surgery 14(4): 593-599. |
| 3 | Excluded - No lap, open, or vaginal comparison cohort | Hutchins, J., D. Delaney, R. I. Vogel, R. G. Ghebre, L. S. Downs, Jr., L. Carson, S. Mullany, D. Teoh and M. A. Geller (2015). "Ultrasound guided subcostal transversus abdominis plane (TAP) infiltration with liposomal bupivacaine for patients undergoing robotic assisted hysterectomy: A prospective randomized controlled study." Gynecol Oncol 138(3): 609-613. |
| 4 | Excluded - No lap, open, or vaginal comparison cohort | Landeen, L. B., E. M. Hultgren, T. M. Kapsch and P. W. Mallory (2016). "Vaginal cuff dehiscence: a randomized trial comparing robotic vaginal cuff closure methods." Journal of Robotic Surgery 10(4): 1-5. |
| 5 | Excluded - No lap, open, or vaginal comparison cohort | Lombardi, T. M., B. S. Kahn, L. J. Tsai, J. M. Waalen and N. Wachi (2019). "Preemptive Oral Compared With Intravenous Acetaminophen for Postoperative Pain After Robotic-Assisted Laparoscopic Hysterectomy: A Randomized Controlled Trial." Obstetrics and gynecology 134(6): 1293-1297. |
| 6 | Excluded - No lap, open, or vaginal comparison cohort | Lyapis, A., A. Ulrich, R. LaMonica, C. L. Kuo, L. Kaye and D. Luciano (2017). "Does the Difference in Fascial Closure Technique Affect Post-Operative Pain?" J Minim Invasive Gynecol. |
| 7 | Excluded - No lap, open, or vaginal comparison cohort | Manoucheri, E., N. Fuchs-Weizman, S. L. Cohen, K. C. Wang and J. Einarsson (2014). "MAUDE: Analysis of Robotic-Assisted Gynecologic Surgery." Journal of Minimally Invasive Gynecology 21(4): 592-595. |
| 8 | Excluded - No lap, open, or vaginal comparison cohort | Shields, K. M., L. E. Minion, L. J. Willmott, D. A. Sumner and B. J. Monk (2015). "Ten-Year Food and Drug Administration Reporting on Robotic Complications in Gynecologic Surgery." Journal of Gynecologic Surgery 31(6): 331-335. |
| 9 | Excluded - No lap, open, or vaginal comparison cohort | Steck-Bayat, K. P., S. Henderson, A. G. Aguirre, R. B. Smith, N. M. Mahnert, R. D. Gerkin and J. Mourad (2019). "Prospective randomized controlled trial comparing cephalad migration in robotic gynecologic surgery using egg-crate foam versus the Pink Pad®." Journal of Robotic Surgery 14(2): 343-347. |
| 10 | Excluded - No lap, open, or vaginal comparison cohort | Tillmanns, T. D., A. Mabe, M. A. Ulm, D. Lee, P. Lowe and S. Kumar (2016). "Vaginal Cuff Closure in Robotic Hysterectomy: A Randomized Controlled Trial Comparing Barbed Versus Standard Suture." Journal of Gynecologic Surgery 32(4): 215-219. |
| 11 | Excluded - No lap, open, or vaginal comparison cohort | Torup, H., M. Bogeskov, E. G. Hansen, C. Palle, J. Rosenberg, A. U. Mitchell, P. L. Petersen, O. Mathiesen, J. B. Dahl and A. M. Moller (2015). "Transversus abdominis plane (TAP) block after robot-assisted laparoscopic hysterectomy: a randomised clinical trial." Acta Anaesthesiol Scand 59(7): 928-935. |
| 12 | Excluded - No lap, open, or vaginal comparison cohort | Tsafrir, Z., M. Palmer, M. Dahlman, A. K. Nawfal, J. Aoun, A. Taylor, J. Fisher, E. Theoharis and D. Eisenstein (2017). "Long-term outcomes for different vaginal cuff closure techniques in robotic-assisted laparoscopic hysterectomy: A randomized controlled trial." Eur J Obstet Gynecol Reprod Biol 210: 7-12. |
| 1 | Excluded - Not in English | Johanson, M. L., M. Dögl and M. Lieng (2020). "Hysterectomy in Norway 2008-18." Tidsskrift for den Norske laegeforening : tidsskrift for praktisk medicin, ny raekke 140(14). |
| 1 | Excluded - Single-port | Kliethermes, C., K. Blazek, K. Ali, J. B. Nijjar, S. Kliethermes and X. Guan (2017). "Postoperative Pain After Single-Site Versus Multiport Hysterectomy." Jsls 21(4). |
| 1 | Excluded - No stratified analysis | Agrawal, S., L. Chen, A. I. Tergas, J. Y. Hou, C. M. St. Clair, C. V. Ananth, A. I. Neugut, D. L. Hershman and J. D. Wright (2018). "Characteristics associated with prolonged length of stay after hysterectomy for benign gynecologic conditions." American Journal of Obstetrics and Gynecology 219(1): 89.e81-89.e15. |
| 2 | Excluded - No stratified analysis | Barber, E. L., E. C. Rossi, A. Alexander, K. Bilimoria and M. A. Simon (2018). "Benign hysterectomy performed by gynecologic oncologists: Is selection bias altering our ability to measure surgical quality?" Gynecol Oncol 151(1): 141-144. |
| 3 | Excluded - No stratified analysis | Barber, E. L., R. M. Polan, A. E. Strohl, M. T. Siedhoff and D. L. Clarke-Pearson (2019). "Cystoscopy at the Time of Hysterectomy for Benign Indications and Delayed Lower Genitourinary Tract Injury." Obstet Gynecol 133(5): 888-895. |
| 4 | Excluded - No stratified analysis | Barron, K. I., G. M. Lamvu, R. C. Schmidt, M. Fisk, E. Blanton and I. Patanwala (2017). "Wound Infiltration With Extended-Release Versus Short-Acting Bupivacaine Before Laparoscopic Hysterectomy: A Randomized Controlled Trial." J Minim Invasive Gynecol 24(2): 286-292. |
| 5 | Excluded - No stratified analysis | Benson, C. R., S. Thompson, G. Li, D. Asafu-Adjei and S. B. Brandes (2020). "Bladder and ureteral injuries during benign hysterectomy: an observational cohort analysis in New York State." World J Urol 38(8): 2049-2054. |
| 6 | Excluded - No stratified analysis | Bougie, O., S. S. Singh, I. Chen and E. P. McCarthy (2019). "Relationship between Race/Ethnicity and Hysterectomy Outcomes for Benign Gynecologic Conditions." Journal of Minimally Invasive Gynecology 26(3): 456-462. |
| 7 | Excluded - No stratified analysis | Callegari, L. S., J. G. Katon, K. E. Gray, K. Doll, S. Pauk, K. E. Lynch, U. S. Uchendu, L. Zephyrin and C. Gardella (2019). "Associations between Race/Ethnicity, Uterine Fibroids, and Minimally Invasive Hysterectomy in the VA Healthcare System." Womens Health Issues 29(1): 48-55. |
| 8 | Excluded - No stratified analysis | Catanzarite, T., S. Saha, M. A. Pilecki, J. Y. Kim and M. P. Milad (2015). "Longer Operative Time During Benign Laparoscopic and Robotic Hysterectomy Is Associated With Increased 30-Day Perioperative Complications." J Minim Invasive Gynecol 22(6): 1049-1058. |
| 9 | Excluded - No stratified analysis | Chung, G., P. Hinoul, P. Coplan and A. Yoo (2021). "Trends in the diffusion of robotic surgery in prostate, uterus, and colorectal procedures: a retrospective population-based study." J Robot Surg 15(2): 275-291. |
| 10 | Excluded - No stratified analysis | Cohen, S. L., M. O. Ajao, N. V. Clark, A. F. Vitonis and J. I. Einarsson (2017). "Outpatient Hysterectomy Volume in the United States." Obstetrics and Gynecology 130(1): 130-137. |
| 11 | Excluded - No stratified analysis | Cohen, S. L., S. N. Morris, D. N. Brown, J. A. Greenberg, B. W. Walsh, A. R. Gargiulo, K. B. Isaacson, K. N. Wright, S. S. Srouji, R. M. Anchan, A. B. Vogell and J. I. Einarsson (2016). "Contained tissue extraction using power morcellation: prospective evaluation of leakage parameters." American journal of obstetrics and gynecology 214(2): 257.e251-256. |
| 12 | Excluded - No stratified analysis | Collins, G. G., J. A. Gadzinski, G. D. Fitzgerald, J. Sheran, S. Wagner, S. Edelstein and E. R. Mueller (2016). "Surgical Pain Control With Ropivacaine by Atomized Delivery (Spray): A Randomized Controlled Trial." J Minim Invasive Gynecol 23(1): 40-45. |
| 13 | Excluded - No stratified analysis | Cope, A. G., M. M. Wetzstein, K. C. Mara, S. K. Laughlin-Tommaso, N. S. Warner and T. L. Burnett (2021). "Abdominal ice following laparoscopic hysterectomy: a randomized controlled trial." J Minim Invasive Gynecol 28(2): 342-350 e342. |
| 14 | Excluded - No stratified analysis | Doll, K. M., S. B. Dusetzina and W. Robinson (2016). "Trends in Inpatient and Outpatient Hysterectomy and Oophorectomy Rates Among Commercially Insured Women in the United States, 2000-2014." JAMA Surg 151(9): 876-877. |
| 15 | Excluded - No stratified analysis | Driessen, S. R. C., E. M. Sandberg, S. P. Rodrigues, E. W. van Zwet and F. W. Jansen (2017). "Identification of risk factors in minimally invasive surgery: a prospective multicenter study." Surgical Endoscopy and Other Interventional Techniques 31(6): 2467-2473. |
| 16 | Excluded - No stratified analysis | Farag, S., L. Rosen and C. Ascher-Walsh (2018). "Comparison of the Memory Foam Pad Versus the Bean Bag with Shoulder Braces in Preventing Patient Displacement during Gynecologic Laparoscopic Surgery." J Minim Invasive Gynecol 25(1): 153-157. |
| 17 | Excluded - No stratified analysis | Farag, S., P. F. Padilla, K. A. Smith, S. E. Zimberg and M. L. Sprague (2021). "Postoperative Urinary Retention Rates after Autofill versus Backfill Void Trial following Total Laparoscopic Hysterectomy: A Randomized Controlled Trial." J Minim Invasive Gynecol 28(4): 829-837. |
| 18 | Excluded - No stratified analysis | Gutierrez, M. M., J. D. Pedroso, K. W. Volker, D. L. Howard and S. D. McCarus (2017). "The McCarus-Volker ForniSee(R): A Novel Trans-illuminating Colpotomy Device and Uterine Manipulator for Use in Conventional and Robotic-Assisted Laparoscopic Hysterectomy." Surg Technol Int 30: 191-196. |
| 19 | Excluded - No stratified analysis | Hesselman, S., L. Bergman, U. Hogberg and M. Jonsson (2018). "Risk of fistula formation and long-term health effects after a benign hysterectomy complicated by organ injury: A population-based register study." Acta Obstet Gynecol Scand 97(12): 1463-1470. |
| 20 | Excluded - No stratified analysis | Hill, A. M., K. M. Davis, L. Clark-Donat, L. M. Hammons, M. Azodi and D. A. Silasi (2017). "The Effect of Vertical Versus Horizontal Vaginal Cuff Closure on Vaginal Length After Laparoscopic Hysterectomy." J Minim Invasive Gynecol 24(1): 108-113. |
| 21 | Excluded - No stratified analysis | Hutchins, J., P. Argenta, A. Berg, J. Habeck, A. Kaizer and M. A. Geller (2019). "Ultrasound-guided subcostal transversus abdominis plane block with liposomal bupivacaine compared to bupivacaine infiltration for patients undergoing robotic-assisted and laparoscopic hysterectomy: a prospective randomized study." J Pain Res 12: 2087-2094. |
| 22 | Excluded - No stratified analysis | Katon, J. G., A. S. Bossick, K. M. Doll, J. Fortney, K. E. Gray, P. Hebert, K. E. Lynch, E. W. Ma, D. L. Washington, L. Zephyrin and L. S. Callegari (2019). "Contributors to racial disparities in minimally invasive hysterectomy in the US Department of Veterans Affairs." Medical Care 57(12): 930-936. |
| 23 | Excluded - No stratified analysis | Katon, J. G., L. S. Callegari, A. S. Bossick, J. Fortney, M. R. Gerber, K. Lehavot, K. E. Lynch, E. Ma, R. Smith, E. Tartaglione and K. E. Gray (2020). "Association of Depression and Post-Traumatic Stress Disorder with Receipt of Minimally Invasive Hysterectomy for Uterine Fibroids: Findings from the U.S. Department of Veterans Affairs." Women's Health Issues 30(5): 359-365. |
| 24 | Excluded - No stratified analysis | Keil, D. S., L. D. Schiff, E. T. Carey, J. K. Moulder, A. M. Goetzinger, S. M. Patidar, L. M. Hance, L. M. Kolarczyk, R. S. Isaak, P. D. Strassle and J. W. Schoenherr (2018). "Predictors of Admission After the Implementation of an Enhanced Recovery After Surgery Pathway for Minimally Invasive Gynecologic Surgery." Anesth Analg. |
| 25 | Excluded - No stratified analysis | Kelly, E. C., J. Winick-Ng, J. A. McClure, T. Peart, Q. Chou, B. MacMillan, Y. Leong, A. Vilos, G. Vilos, B. Welk and J. McGee (2019). "Hysterectomy in Ontario: A Population-Based Study of Outcomes and Complications in Minimally Invasive Compared with Abdominal Approaches." J Obstet Gynaecol Can 41(8): 1168-1176. |
| 26 | Excluded - No stratified analysis | Lauterbach, R., M. Joseph, Z. Haklai, L. Gil and L. Lowenstein (2019). "Geographic variation of hysterectomy rates in the Israeli health care system during the years 2007-2016." Isr J Health Policy Res 8(1): 52. |
| 27 | Excluded - No stratified analysis | McNanley, A., M. Perevich, C. Glantz, E. E. Duecy, M. K. Flynn and G. Buchsbaum (2012). "Bowel function after minimally invasive urogynecologic surgery: a prospective randomized controlled trial." Female Pelvic Med Reconstr Surg 18(2): 82-85. |
| 28 | Excluded - No stratified analysis | Mehta, A., T. Xu, S. Hutfless, M. A. Makary, A. K. Sinno, E. J. Tanner, 3rd, R. L. Stone, K. Wang and A. N. Fader (2017). "Patient, surgeon, and hospital disparities associated with benign hysterectomy approach and perioperative complications." Am J Obstet Gynecol 216(5): 497 e491-497 e410. |
| 29 | Excluded - No stratified analysis | Mikhail, E., N. Tamhane, P. Sarkar, E. Sappenfield, J. P. Tanner and A. N. Imudia (2019). "Laparoscopic Entry Technique Using a Veress Needle Insertion with and without Concomitant CO2 Insufflation: A Randomized Controlled Trial." Journal of Minimally Invasive Gynecology 26(7): 1383-1388. |
| 30 | Excluded - No stratified analysis | Mikhail, E., P. Sarkar, M. Moucharite and S. Hart (2018). "The Association Between Hospital Surgical Volume and the Uptake of Minimally Invasive Surgical Approach and Outpatient Setting for Hysterectomy." Surg Technol Int 33. |
| 31 | Excluded - No stratified analysis | Misal, M., M. Girardo and M. N. Wasson (2021). "Surgical Decision Regret in Women Pursuing Surgery for Endometriosis or Chronic Pelvic Pain." J Minim Invasive Gynecol 28(7): 1343-1350. |
| 32 | Excluded - No stratified analysis | Moawad, G., P. Tyan, C. Marfori, E. Abi Khalil and D. Park (2019). "Effect of postoperative partial bladder filling after minimally invasive hysterectomy on postanesthesia care unit discharge and cost: a single-blinded, randomized controlled trial." Am J Obstet Gynecol 220(4): 367 e361-367 e367. |
| 33 | Excluded - No stratified analysis | Packiam, V. T., A. J. Cohen, J. J. Pariser, C. U. Nottingham, S. F. Faris and G. T. Bales (2016). "The Impact of Minimally Invasive Surgery on Major Iatrogenic Ureteral Injury and Subsequent Ureteral Repair During Hysterectomy: A National Analysis of Risk Factors and Outcomes." Urology 98: 183-188. |
| 34 | Excluded - No stratified analysis | Schiff, L., G. Wegienka, R. Sangha and D. Eisenstein (2014). "Is cervix removal associated with patient-centered outcomes of pain, dyspareunia, well-being and satisfaction after laparoscopic hysterectomy?" Arch Gynecol Obstet. |
| 35 | Excluded - No stratified analysis | Schmitt, J. J., M. V. Baker, J. A. Occhino, M. E. McGree, A. L. Weaver, J. N. Bakkum-Gamez, S. C. Dowdy, K. S. Pasupathy and J. B. Gebhart (2020). "Prospective Implementation and Evaluation of a Decision-Tree Algorithm for Route of Hysterectomy." Obstetrics and gynecology 135(4): 761-769. |
| 36 | Excluded - No stratified analysis | Settnes, A., M. FinkTopsoee, C. Moeller, M. Dueholm, T. I. Kopp, C. Norrbom, S. C. Rasmussen, P. A. Froeslev, A. Joergensen, E. Dreisler and H. Gimbel (2019). "Reduced complications following implementation of laparoscopic hysterectomy: A Danish population-based cohort study of minimally invasive benign gynecologic surgery 2004-2018." J Minim Invasive Gynecol 27(6): 1344-1353 e1343. |
| 37 | Excluded - No stratified analysis | Sheyn, D., S. Mahajan, M. Billow, A. Fleary, E. Hayashi and S. A. El-Nashar (2017). "Geographic Variance of Cost Associated With Hysterectomy." Obstetrics and Gynecology 129(5): 844-853. |
| 38 | Excluded - No stratified analysis | Shih, Y. T., C. Shen and J. C. Hu (2017). "Do Robotic Surgical Systems Improve Profit Margins? A Cross-Sectional Analysis of California Hospitals." Value Health 20(8): 1221-1225. |
| 39 | Excluded - No stratified analysis | Skinner, B. D., N. S. Kamdar, N. Mahnert, C. S. Lim, A. J. Mullard, D. A. Campbell, S. As-Sanie and D. M. Morgan (2016). "A Favorability Score for Vaginal Hysterectomy in a Statewide Collaborative." J Minim Invasive Gynecol 23(7): 1146-1151. |
| 40 | Excluded - No stratified analysis | Suidan, R. S., W. He, C. C. Sun, H. Zhao, N. D. Fleming, P. T. Ramirez, P. T. Soliman, S. N. Westin, K. H. Lu, S. H. Giordano and L. A. Meyer (2017). "Impact of body mass index and operative approach on surgical morbidity and costs in women with endometrial carcinoma and hyperplasia." Gynecologic Oncology 145(1): 55-60. |
| 41 | Excluded - No stratified analysis | Till, S. R., D. M. Morgan, A. A. Bazzi, M. D. Pearlman, Z. Abdelsattar, D. A. Campbell and S. Uppal (2017). "Reducing Surgical Site Infections after Hysterectomy: Metronidazole plus cefazolin compared to cephalosporin alone." Am J Obstet Gynecol. |
| 42 | Excluded - No stratified analysis | Ulm, M. A., A. C. ElNaggar and T. D. Tillmanns (2018). "Celecoxib versus ketorolac following robotic hysterectomy for the management of postoperative pain: An open-label randomized control trial." Gynecologic Oncology 151(1): 124-128. |
| 43 | Excluded - No stratified analysis | Wallis, C. J. D., S. Peltz, J. Byrne, J. Kroft, P. Karanicolas, N. Coburn, A. B. Nathens, R. K. Nam, J. Hallet and R. Satkunasivam (2017). "Peripheral nerve injury during abdominal-pelvic surgery: Analysis of the National Surgical Quality Improvement Program database." American Surgeon 83(11): 1214-1219. |
| 44 | Excluded - No stratified analysis | Washburn, E. E., S. L. Cohen, E. Manoucheri, R. K. Zurawin and J. I. Einarsson (2014). "Trends in reported resident surgical experience in hysterectomy." J Minim Invasive Gynecol 21(6): 1067-1070. |
| 45 | Excluded - No stratified analysis | Wechter, M. E., R. M. Kho, A. H. Chen, J. F. Magrina and P. D. Pettit (2013). "Preventing slide in Trendelenburg position: Randomized trial comparing foam and gel pads." Journal of Robotic Surgery 7(3): 267-271. |
| 46 | Excluded - No stratified analysis | Wen, X., S. Kogut, H. Aroke, L. Taylor and K. A. Matteson (2020). "Chronic opioid use in women following hysterectomy: Patterns and predictors." Pharmacoepidemiology and Drug Safety 29(4): 493-503. |
| 47 | Excluded - No stratified analysis | Wright, J. D., A. I. Tergas, W. M. Burke, R. R. Cui, C. V. Ananth, L. Chen and D. L. Hershman (2014). "Uterine pathology in women undergoing minimally invasive hysterectomy using morcellation." JAMA 312(12): 1253-1255. |
| 48 | Excluded - No stratified analysis | Yusuf, F., S. Leeder and A. Wilson (2016). "Recent estimates of the incidence of hysterectomy in New South Wales and trends over the past 30 years." Aust N Z J Obstet Gynaecol 56(4): 420-425. |
| 49 | Excluded - No stratified analysis | Zaritsky, E., L. Y. Tucker, R. Neugebauer, T. Chou, T. Flanagan, A. J. Walter and T. Raine-Bennett (2017). "Minimally Invasive Hysterectomy and Power Morcellation Trends in a West Coast Integrated Health System." Obstet Gynecol 129(6): 996-1005. |
| 1 | Excluded - Benign Hysterectomy Mixed | Anderson, J. E., D. C. Chang, J. K. Parsons and M. A. Talamini (2012). "The first national examination of outcomes and trends in robotic surgery in the United States." J Am Coll Surg 215(1): 107-114; discussion 114-106. |
| 2 | Excluded - Benign Hysterectomy Mixed | Dalsgaard, T., M. D. Jensen, D. Hartwell, B. J. Mosgaard, A. Jorgensen and B. R. Jensen (2018). "Robotic Surgery Is Less Physically Demanding Than Laparoscopic Surgery: Paired Cross Sectional Study." Ann Surg. |
| 3 | Excluded - Benign Hysterectomy Mixed | DeStephano, C. C., S. P. Gajarawala, M. Espinal, M. G. Heckman, E. R. Vargas and M. A. Robertson (2019). "Discharge Readiness after Robotic and Laparoscopic Hysterectomy." J Minim Invasive Gynecol 26(5): 910-918. |
| 4 | Excluded - Benign Hysterectomy Mixed | El Hachem, L., U. C. Acholonu, Jr. and F. R. Nezhat (2013). "Postoperative pain and recovery after conventional laparoscopy compared with robotically assisted laparoscopy." Obstetrics and Gynecology 121(3): 547-553. |
| 5 | Excluded - Benign Hysterectomy Mixed | Harris, J. A., S. Uppal, N. Kamdar, C. W. Swenson, D. Campbell and D. M. Morgan (2017). "A retrospective cohort study of hemostatic agent use during hysterectomy and risk of post-operative complications." Int J Gynaecol Obstet 136(2): 232-237. |
| 6 | Excluded - Benign Hysterectomy Mixed | Khorgami, Z., W. T. Li, T. N. Jackson, C. A. Howard and G. M. Sclabas (2019). "The cost of robotics: an analysis of the added costs of robotic-assisted versus laparoscopic surgery using the National Inpatient Sample." Surg Endosc 33(7): 2217-2221. |
| 7 | Excluded - Benign Hysterectomy Mixed | Kurt, G., V. W. Loerzel, R. B. Hines, K. Tavasci, S. Galura, S. Ahmad and R. W. Holloway (2018). "Learning Needs of Women Who Undergo Robotic Versus Open Gynecologic Surgery." J Obstet Gynecol Neonatal Nurs. |
| 8 | Excluded - Benign Hysterectomy Mixed | Pasic, R. P., J. A. Rizzo, H. Fang, S. Ross, M. Moore and C. Gunnarsson (2010). "Comparing robot-assisted with conventional laparoscopic hysterectomy: impact on cost and clinical outcomes." J Minim Invasive Gynecol 17(6): 730-738. |
| 9 | Excluded - Benign Hysterectomy Mixed | Ruiz, M. P., L. Chen, J. Y. Hou, A. I. Tergas, C. M. St Clair, C. V. Ananth, A. I. Neugut, D. L. Hershman and J. D. Wright (2018). "Effect of Minimum-Volume Standards on Patient Outcomes and Surgical Practice Patterns for Hysterectomy." Obstet Gynecol 132(5): 1229-1237. |
| 10 | Excluded - Benign Hysterectomy Mixed | Ruiz, M. P., L. Chen, J. Y. Hou, A. I. Tergas, C. M. St Clair, C. V. Ananth, A. I. Neugut, D. L. Hershman and J. D. Wright (2018). "Outcomes of Hysterectomy Performed by Very Low-Volume Surgeons." Obstet Gynecol 131(6): 981-990. |
| 11 | Excluded - Benign Hysterectomy Mixed | Stewart, C. L., S. Dumitra, C. Nota, P. H. G. Ituarte, L. G. Melstrom, Y. Woo, G. Singh, Y. Fong, H. Nathan and S. G. Warner (2019). "Hospital factors strongly influence robotic use in general surgery." Surgery 166(5): 867-872. |
| 12 | Excluded - Benign Hysterectomy Mixed | Turchetti, G., F. Pierotti, I. Palla, S. Manetti, C. Freschi, V. Ferrari and A. Cuschieri (2016). "Comparative health technology assessment of robotic-assisted, direct manual laparoscopic and open surgery: a prospective study." Surgical Endoscopy and Other Interventional Techniques: 1-9. |
| 13 | Excluded - Benign Hysterectomy Mixed | Wijk, L., O. Ljungqvist and K. Nilsson (2019). "Female sex hormones in relation to insulin resistance after hysterectomy: A pilot study." Clin Nutr 38(6): 2721-2726. |
| 14 | Excluded - Benign Hysterectomy Mixed | Wijk, L., K. Nilsson and O. Ljungqvist (2018). "Metabolic and inflammatory responses and subsequent recovery in robotic versus abdominal hysterectomy: A randomised controlled study." Clinical Nutrition 37(1): 99-106. |
| 15 | Excluded - Benign Hysterectomy Mixed | Wright, J. D., A. I. Tergas, J. Y. Hou, W. M. Burke, L. Chen, J. C. Hu, A. I. Neugut, C. V. Ananth and D. L. Hershman (2016). "Effect of Regional Hospital Competition and Hospital Financial Status on the Use of Robotic-Assisted Surgery." JAMA Surg. |
| 16 | Excluded - Benign Hysterectomy Mixed | Zechmeister, J. R., T. L. Pua, L. R. Boyd, S. V. Blank, J. P. Curtin and B. Pothuri (2015). "A prospective comparison of postoperative pain and quality of life in robotic assisted vs conventional laparoscopic gynecologic surgery." American Journal of Obstetrics and Gynecology 212(2): 194.e191-194.e197. |
| 1 | Excluded - No outcomes of interest | Bush, A. J., S. N. Morris, F. H. Millham and K. B. Isaacson (2011). "Women's Preferences for Minimally Invasive Incisions." Journal of Minimally Invasive Gynecology 18(5): 640-643. |
| 2 | Excluded - No outcomes of interest | Desai, V. B., X. M. Guo, L. Fan, J. D. Wright and X. Xu (2017). "Inpatient Laparoscopic Hysterectomy in the United States: Trends and Factors Associated With Approach Selection." J Minim Invasive Gynecol 24(1): 151-158 e151. |
| 3 | Excluded - No outcomes of interest | Desai, V. B. and X. Xu (2015). "An update on inpatient hysterectomy routes in the United States." American Journal of Obstetrics and Gynecology 213(5): 742-743. |
| 4 | Excluded - No outcomes of interest | Katon, J. G., K. Gray, L. Callegari, C. Gardella, C. Gibson, E. Ma, K. E. Lynch and L. Zephyrin (2017). "Trends in hysterectomy rates among women veterans in the US Department of Veterans Affairs." Am J Obstet Gynecol. |
| 5 | Excluded - No outcomes of interest | Laursen, K. R., V. B. Hyldgard, P. T. Jensen and R. Sogaard (2018). "Health care cost consequences of using robot technology for hysterectomy: a register-based study of consecutive patients during 2006-2013." J Robot Surg 12(2): 283-294. |
| 6 | Excluded - No outcomes of interest | Marcus, H. J., A. Hughes-Hallett, C. J. Payne, T. P. Cundy, D. Nandi, G. Z. Yang and A. Darzi (2017). "Trends in the diffusion of robotic surgery: A retrospective observational study." Int J Med Robot. |
| 7 | Excluded - No outcomes of interest | Moawad, G., E. Liu, C. Song and A. Z. Fu (2017). "Movement to outpatient hysterectomy for benign indications in the United States, 2008–2014." PLoS ONE 12(11): e0188812. |
| 8 | Excluded - No outcomes of interest | Obermair, H. M. and E. J. Borg (2019). "Salpingectomy at the time of hysterectomy for benign gynaecological disease: A comparison of surgical approaches." Aust N Z J Obstet Gynaecol 59(5): 725-729. |
| 9 | Excluded - No outcomes of interest | Sampat, A., I. Parakati, R. Kunnavakkam, D. B. Glick, N. K. Lee, M. Tenney, S. Eggener and S. Roth (2015). "Corneal abrasion in hysterectomy and prostatectomy: role of laparoscopic and robotic assistance." Anesthesiology 122(5): 994-1001. |
| 10 | Excluded - No outcomes of interest | Smith, A. J. B., A. AlAshqar, K. F. Chaves and M. A. Borahay (2020). "Association of demographic, clinical, and hospital-related factors with use of robotic hysterectomy for benign indications: A national database study." International Journal of Medical Robotics and Computer Assisted Surgery 16(4): e2107. |
| 11 | Excluded - No outcomes of interest | Wright, J. D., C. V. Ananth, A. I. Tergas, T. J. Herzog, W. M. Burke, S. N. Lewin, Y. S. Lu, A. I. Neugut and D. L. Hershman (2014). "An economic analysis of robotically assisted hysterectomy." Obstet Gynecol 123(5): 1038-1048. |
| 1 | Excluded - redundant patient population with Martinez-Maestre 2014 | Martínez-Maestre, M. A., L. M. Melero-Cortés, P. J. Coronado, C. González-Cejudo, N. García-Agua, A. J. García-Ruíz and F. Jódar-Sánchez (2019). "Long term COST-minimization analysis of robot-assisted hysterectomy versus conventional laparoscopic hysterectomy." Health Economics Review 9(1): 18. |
| 1 | Included | Billfeldt, N. K., C. Borgfeldt, H. Lindkvist, J. H. Stjerndahl and M. Ankardal (2018). "A Swedish population-based evaluation of benign hysterectomy, comparing minimally invasive and abdominal surgery." European Journal of Obstetrics Gynecology and Reproductive Biology 222: 113-118. |
| 2 | Included | Brunes, M., U. Johannesson, H. Habel, M. W. Soderberg and M. Ek (2021). "Effects of obesity on peri- and postoperative outcomes in patients undergoing robotic vs. conventional hysterectomy." J Minim Invasive Gynecol 28(2): 228-236. |
| 3 | Included | Carbonnel, M., H. Abbou, H. T. N'Guyen, S. Roy, G. Hamdi, A. Jnifen and J. M. Ayoubi (2013). "Robotically assisted hysterectomy versus vaginal hysterectomy for benign disease: A prospective study." Minimally Invasive Surgery 2013: 429105. |
| 4 | Included | Cohen, S. L., A. F. Vitonis and J. I. Einarsson (2014). "Updated hysterectomy surveillance and factors associated with minimally invasive hysterectomy." JSLS 18(3). |
| 5 | Included | Dandolu, V. and P. Pathak (2018). "Health resource utilization and costs during the first 90 days following robot-assisted hysterectomy." Int Urogynecol J 29(6): 865-872. |
| 6 | Included | Deimling, T. A., J. L. Eldridge, K. A. Riley, A. R. Kunselman and G. J. Harkins (2017). "Randomized controlled trial comparing operative times between standard and robot-assisted laparoscopic hysterectomy." Int J Gynaecol Obstet 136(1): 64-69. |
| 7 | Included | Dubeshter, B., C. Angel, E. Toy, S. Thomas and J. C. Glantz (2013). "Current role of robotic hysterectomy." Journal of Gynecologic Surgery 29(4): 174-178. |
| 8 | Included | Elessawy, M., S. Schneekloth, V. Günther, N. Maass, L. Mettler and I. Alkatout (2020). "Postoperative telephone-based questionnaire on quality of life after robotic-assisted laparoscopic hysterectomy versus conventional total laparoscopic hysterectomy." Journal of Clinical Medicine 9(9): 1-13. |
| 9 | Included | Friedman, B., G. I. Barbash, S. A. Glied and C. A. Steiner (2016). "Hospital Revisits Within 30 Days After Conventional and Robotically Assisted Hysterectomy." Med Care 54(3): 311-318. |
| 10 | Included | Hart, S., L. Hashemi and C. J. Sobolewski (2013). "Effect of a disposable automated suturing device on cost and operating room time in benign total laparoscopic hysterectomy procedures." Jsls 17(4): 508-516. |
| 11 | Included | Herrinton, L. J., T. Raine-Bennett, L. Liu, S. E. Alexeeff, W. Ramos and B. Suh-Burgmann (2020). "Outcomes of Robotic Hysterectomy for Treatment of Benign Conditions: Influence of Patient Complexity." The Permanente journal 24. |
| 12 | Included | Lim, C. S., E. L. Mowers, N. Mahnert, B. D. Skinner, N. Kamdar, D. M. Morgan and S. As-Sanie (2016). "Risk Factors and Outcomes for Conversion to Laparotomy of Laparoscopic Hysterectomy in Benign Gynecology." Obstet Gynecol 128(6): 1295-1305. |
| 13 | Included | Lim, P. C., J. T. Crane, E. J. English, R. W. Farnam, D. M. Garza, M. L. Winter and J. L. Rozeboom (2016). "Multicenter analysis comparing robotic, open, laparoscopic, and vaginal hysterectomies performed by high-volume surgeons for benign indications." Int J Gynaecol Obstet 133(3): 359-364. |
| 14 | Included | Lonnerfors, C., P. Reynisson and J. Persson (2015). "A randomized trial comparing vaginal and laparoscopic hysterectomy vs robot-assisted hysterectomy." J Minim Invasive Gynecol 22(1): 78-86. |
| 15 | Included | Luciano, A. A., D. E. Luciano, J. Gabbert and U. Seshadri-Kreaden (2016). "The impact of robotics on the mode of benign hysterectomy and clinical outcomes." Int J Med Robot 12(1): 114-124. |
| 16 | Included | Martinez-Maestre, M. A., P. Gambadauro, C. Gonzalez-Cejudo and R. Torrejon (2014). "Total laparoscopic hysterectomy with and without robotic assistance: a prospective controlled study." Surg Innov 21(3): 250-255. |
| 17 | Included | Ngan, T. Y. T., A. Zakhari, N. Czuzoj-Shulman, T. Tulandi and H. A. Abenhaim (2018). "Laparoscopic and Robotic-Assisted Hysterectomy for Uterine Leiomyomas: A Comparison of Complications and Costs." Journal of Obstetrics and Gynaecology Canada 40(4): 432-439. |
| 18 | Included | Paraiso, M. F., B. Ridgeway, A. J. Park, J. E. Jelovsek, M. D. Barber, T. Falcone and J. I. Einarsson (2013). "A randomized trial comparing conventional and robotically assisted total laparoscopic hysterectomy." Am J Obstet Gynecol 208(5): 368 e361-367. |
| 19 | Included | Pellegrino, A., G. R. Damiani, G. Fachechi, S. Corso, C. Pirovano, C. Trio, M. Villa, D. Turoli and A. Youssef (2017). "Cost analysis of minimally invasive hysterectomy vs open approach performed by a single surgeon in an Italian center." J Robot Surg 11(2): 115-121. |
| 20 | Included | Rosero, E. B., K. A. Kho, G. P. Joshi, M. Giesecke and J. I. Schaffer (2013). "Comparison of robotic and laparoscopic hysterectomy for benign gynecologic disease." Obstet Gynecol 122(4): 778-786. |
| 21 | Included | Sarlos, D., L. Kots, N. Stevanovic, S. von Felten and G. Schar (2012). "Robotic compared with conventional laparoscopic hysterectomy: a randomized controlled trial." Obstet Gynecol 120(3): 604-611. |
| 22 | Included | Swenson, C. W., N. S. Kamdar, J. A. Harris, S. Uppal, D. A. Campbell, Jr. and D. M. Morgan (2016). "Comparison of robotic and other minimally invasive routes of hysterectomy for benign indications." Am J Obstet Gynecol 215(5): 650.e651-650.e658. |
| 23 | Included | Ulubay, M., M. Dede, M. Ozturk, U. Keskin, U. Fidan, I. Alanbay and M. C. Yenen (2016). "Comparison of Robotic-Assisted and Abdominal Hysterectomy with Concomitant Burch Colposuspension." Journal of Gynecologic Surgery 32(2): 119-123. |
| 24 | Included | Wright, J. D., C. V. Ananth, S. N. Lewin, W. M. Burke, Y. S. Lu, A. I. Neugut, T. J. Herzog and D. L. Hershman (2013). "Robotically assisted vs laparoscopic hysterectomy among women with benign gynecologic disease." JAMA 309(7): 689-698. |
