# Supplementary material for: Robotic-assisted benign hysterectomy compared with laparoscopic, vaginal, and open surgery: a systematic review and meta-analysis
Source: J Robot Surg. 2023 Oct 19;17(6):2647–62. doi: 10.1007/s11701-023-01724-6 (PMC10678826; doi:10.1007/s11701-023-01724-6)
Supplement: Supplementary file 3 — Online Resource 3: Newcastle Ottawa Score (NOS). A table with the risk of bias assessment results using the Newcastle Ottawa Score [file 11701_2023_1724_MOESM3_ESM.docx]

Robotic-assisted benign hysterectomy compared with laparoscopic, vaginal, and open surgery: A systematic review and meta-analysis. Journal of Robotic Surgery.

Louis Lenfant^1,2^, Geoffroy Canlorbe^2^, Jérémie Belghiti^2^, Usha Seshadri Kreaden^3^, April E. Hebert^3^, Marianne Nikpayam^2^, Catherine Uzan^2^, Henri Azaïs^2,4*^

1 Sorbonne Université, Department of Urology, Academic Hospital Pitié-Salpêtrière, APHP, F-75013 PARIS, France

2 Department of Surgery and Oncological Gynecology, Pitié-Salpétrière University Hospital, Assistance Publique des Hôpitaux de Paris, Sorbonne University, Paris, France

3 Biostatistics & Global Evidence Management, Intuitive Surgical Inc, Sunnyvale, California

4 Gynecologic and Breast Oncologic Surgery Department, Georges Pompidou European Hospital, APHP. Centre, Université de Paris Cité, Paris, France

*Corresponding author E-mail: henriazais@gmail.com (HA)

Newcastle Ottawa Score (NOS)

|  | **Selection** | | | | **Comparability** | **Outcome** | | |  |
| --- | --- | --- | --- | --- | --- | --- | --- | --- | --- |
| **Author, year** | Exposed cohort truly or somewhat representative of the community | Non-exposed cohort drawn from the same community as exposed cohort | Ascertainment of exposure by secure record or structured interview | Demonstrated outcome of interest not present at start of study | Study controls for baseline characteristics (one star) and/or any additional factors (one star) | Assessment of outcome by independent blind assessment or record linkage | Follow-up enough for outcomes to occur | Complete follow up or ≤20% lost to FU, no different from those followed, loss unlikely to introduce bias | **Total Quality Score** |
| **Billfeldt 2018** | * | * | * | * | ** | * | * |  | 8 |
| **Brunes 2021** | * | * | * | * | ** | * | * | * | 9 |
| **Carbonnel 2013** | * | * | * | * |  | * | * |  | 6 |
| **Cohen 2014** | * | * | * | * | ** | * | * | * | 9 |
| **Dandolu 2018** | * | * | * | * | * | * | * |  | 7 |
| **Dubeshter 2013** | * | * | * | * | ** | * |  |  | 7 |
| **Elessawy 2020** | * | * | * | * | ** | * | * |  | 8 |
| **Friedman 2016** | * | * | * | * | ** | * | * | * | 9 |
| **Hart 2013** | * | * | * | * | * | * | * |  | 7 |
| **Herrinton 2020** | * | * | * | * | ** | * | * | * | 9 |
| **Lim, CS 2016 Risk** | * | * | * | * | ** | * | * | * | 9 |
| **Lim, PC 2016 Multi** | * | * | * | * |  | * | * | * | 7 |
| **Luciano 2016** | * | * | * | * |  | * | * |  | 6 |
| **Martinez-Maestre 2014** | * | * | * | * | * | * | * | * | 8 |
| **Ngan 2018** | * | * | * | * | * | * |  |  | 6 |
| **Pellegrino 2016** | * | * | * | * | ** | * | * |  | 8 |
| **Rosero 2013** | * | * | * | * | ** | * | * |  | 8 |
| **Swenson 2016** | * | * | * | * | ** | * | * |  | 8 |
| **Ulubay 2016** | * | * | * | * |  | * | * |  | 6 |
| **Wright 2013** | * | * | * | * | ** | * | * |  | 8 |
