# Supplementary material for: Robotic-assisted benign hysterectomy compared with laparoscopic, vaginal, and open surgery: a systematic review and meta-analysis
Source: J Robot Surg. 2023 Oct 19;17(6):2647–62. doi: 10.1007/s11701-023-01724-6 (PMC10678826; doi:10.1007/s11701-023-01724-6)
Supplement: Supplementary file 4 — Online Resource 4: Sensitivity analysis limited to RCT and prospective studies. A table with the number of studies and patients included in each analysis of outcomes with RCT or prospective studies, along with the effect size, 90% confidence interval, p-value, and heterogeneity analysis. Bolding indicates significance (p<0.05) [file 11701_2023_1724_MOESM4_ESM.docx]

Robotic-assisted benign hysterectomy compared with laparoscopic, vaginal, and open surgery: A systematic review and meta-analysis. Journal of Robotic Surgery. Louis Lenfant^1,2^, Geoffroy Canlorbe^2^, Jérémie Belghiti^2^, Usha Seshadri Kreaden^3^, April E. Hebert^3^, Marianne Nikpayam^2^, Catherine Uzan^2^, Henri Azaïs^2,4*^

1 Sorbonne Université, Department of Urology, Academic Hospital Pitié-Salpêtrière, APHP, F-75013 PARIS, France

2 Department of Surgery and Oncological Gynecology, Pitié-Salpétrière University Hospital, Assistance Publique des Hôpitaux de Paris, Sorbonne University, Paris, France

3 Biostatistics & Global Evidence Management, Intuitive Surgical Inc, Sunnyvale, California

4 Gynecologic and Breast Oncologic Surgery Department, Georges Pompidou European Hospital, APHP. Centre, Université de Paris Cité, Paris, France

*Corresponding author E-mail: henriazais@gmail.com (HA)

Summary of Sensitivity-Analysis – RCT and prospective studies only

| Meta-Analysis | | | | | | | Heterogeneity | | |
| --- | --- | --- | --- | --- | --- | --- | --- | --- | --- |
| Outcome | | # Studies | Robot n | Comparator n | Effect size [95% CI] | p-value | model | Heterogeneity I^2^ | p-value |
| Robotic vs. Laparoscopic | | | | | | | | | |
| Operative Time, min | | 7 | 377 | 465 | MD: 7-59 [-10.69, 25.87] | 0.42 | **RE** | **92%** | **<0.00001** |
|  | EBL, mL | 3 | 172 | 214 | MD: -18.12 [-49.38, 13.15] | 0.26 | **RE** | **68%** | **0.04** |
|  | Transfusions | 4 | 97 | 309 | RD: 0.0001 [-0.0217, 0.0219] | 0.99 | FE | 0% | 0.83 |
|  | Conversions | 7 | 377 | 465 | RD: -0.02 [-0.03, 0.002] | 0.08 | FE | 0% | 0.49 |
| Intraoperative Complications | | 5 | 287 | 309 | OR: 1.38 [0.70, 2.73] | 0.35 | FE | 0% | 0.76 |
| Postoperative Complications* | | 6 | 351 | 439 | OR: 0.76 [0.44, 1.31] | 0.32 | FE | 43% | 0.12 |
|  | Mortality | 2 | 107 | 153 | RD: 0.00 [-0.02, 0.02] | 1.00 | FE | 0% | 1.00 |
|  | LOS, days | 7 | 377 | 465 | **MD: -0.28 [-0.51, -0.05]** | **0.02** | **RE** | **86%** | **<0.00001** |
|  | Readmissions | 3 | 197 | 238 | RD: -0.02 [-0.05, 0.01] | 0.27 | FE | 22% | 0.28 |
|  | Reoperation | 3 | 143 | 161 | RD: -0.01 [-0.05, 0.02] | 0.47 | FE | 0% | 0.68 |

Footnotes: MD: mean difference, OR: odds ratio, RD: risk difference, RE: random effects model, FE: fixed effects model, CI: confidence interval. Bolding indicates significance (p<0.05). ^a^Postoperative complication rate was extracted preferentially, but papers that reported only one complication rate (sometimes labeled “perioperative”) were also included in the analysis.
