# Supplementary material for: Robotic-assisted benign hysterectomy compared with laparoscopic, vaginal, and open surgery: a systematic review and meta-analysis
Source: J Robot Surg. 2023 Oct 19;17(6):2647–62. doi: 10.1007/s11701-023-01724-6 (PMC10678826; doi:10.1007/s11701-023-01724-6)

Robotic-assisted benign hysterectomy compared with laparoscopic, vaginal, and open surgery: A systematic review and meta-analysis. Journal of Robotic Surgery.

Louis Lenfant^1,2^, Geoffroy Canlorbe^2^, Jérémie Belghiti^2^, Usha Seshadri Kreaden^3^, April E. Hebert^3^, Marianne Nikpayam^2^, Catherine Uzan^2^, Henri Azaïs^2,4*^

1 Sorbonne Université, Department of Urology, Academic Hospital Pitié-Salpêtrière, APHP, F-75013 PARIS, France

2 Department of Surgery and Oncological Gynecology, Pitié-Salpétrière University Hospital, Assistance Publique des Hôpitaux de Paris, Sorbonne University, Paris, France

3 Biostatistics & Global Evidence Management, Intuitive Surgical Inc, Sunnyvale, California

4 Gynecologic and Breast Oncologic Surgery Department, Georges Pompidou European Hospital, APHP. Centre, Université de Paris Cité, Paris, France

*Corresponding author E-mail: henriazais@gmail.com (HA)

Funnel Plots


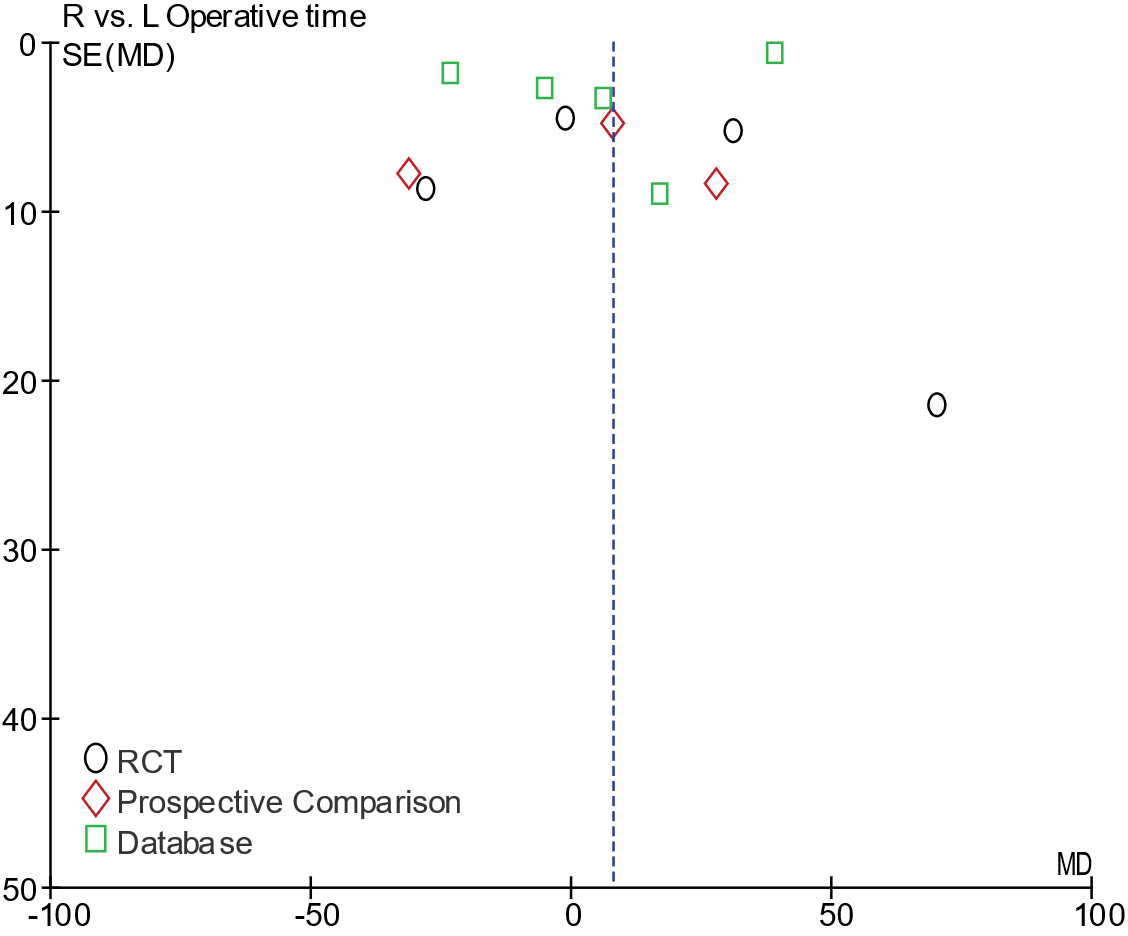

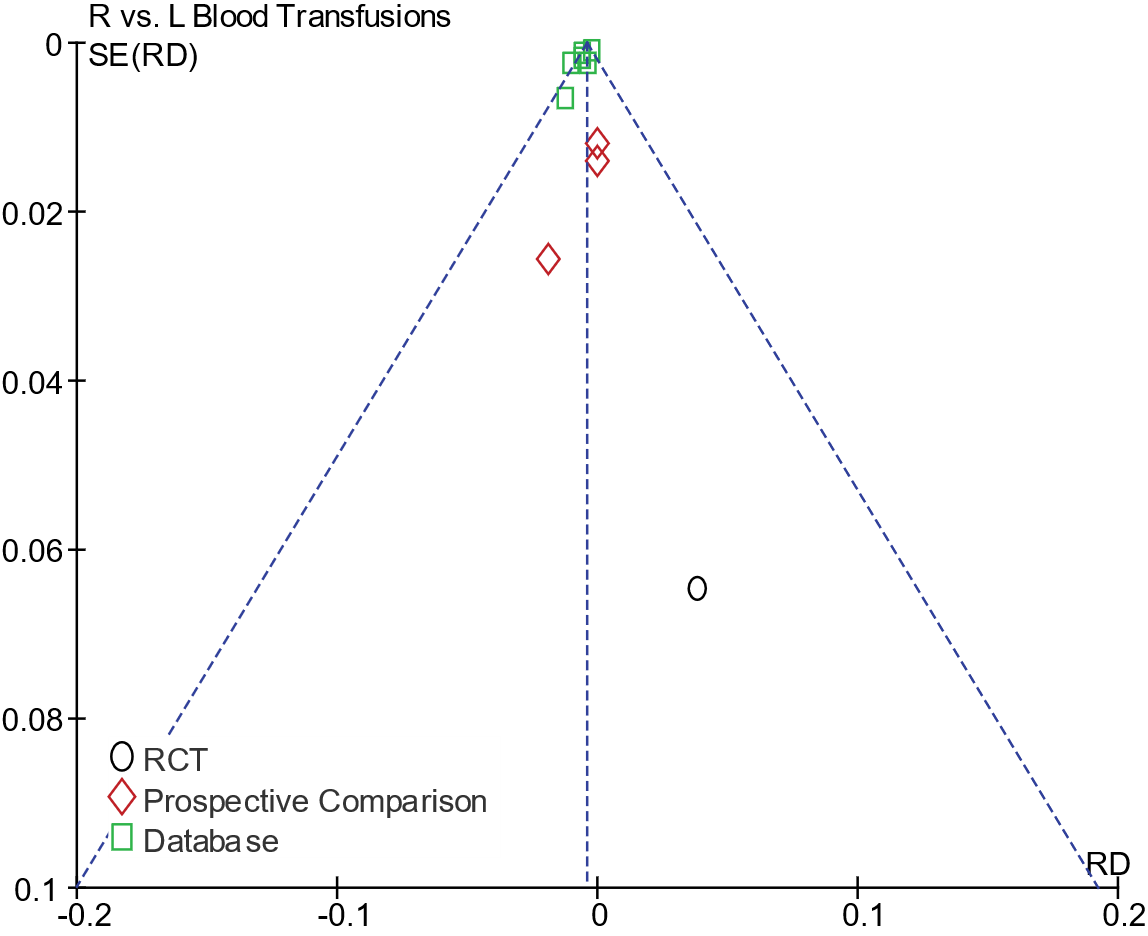


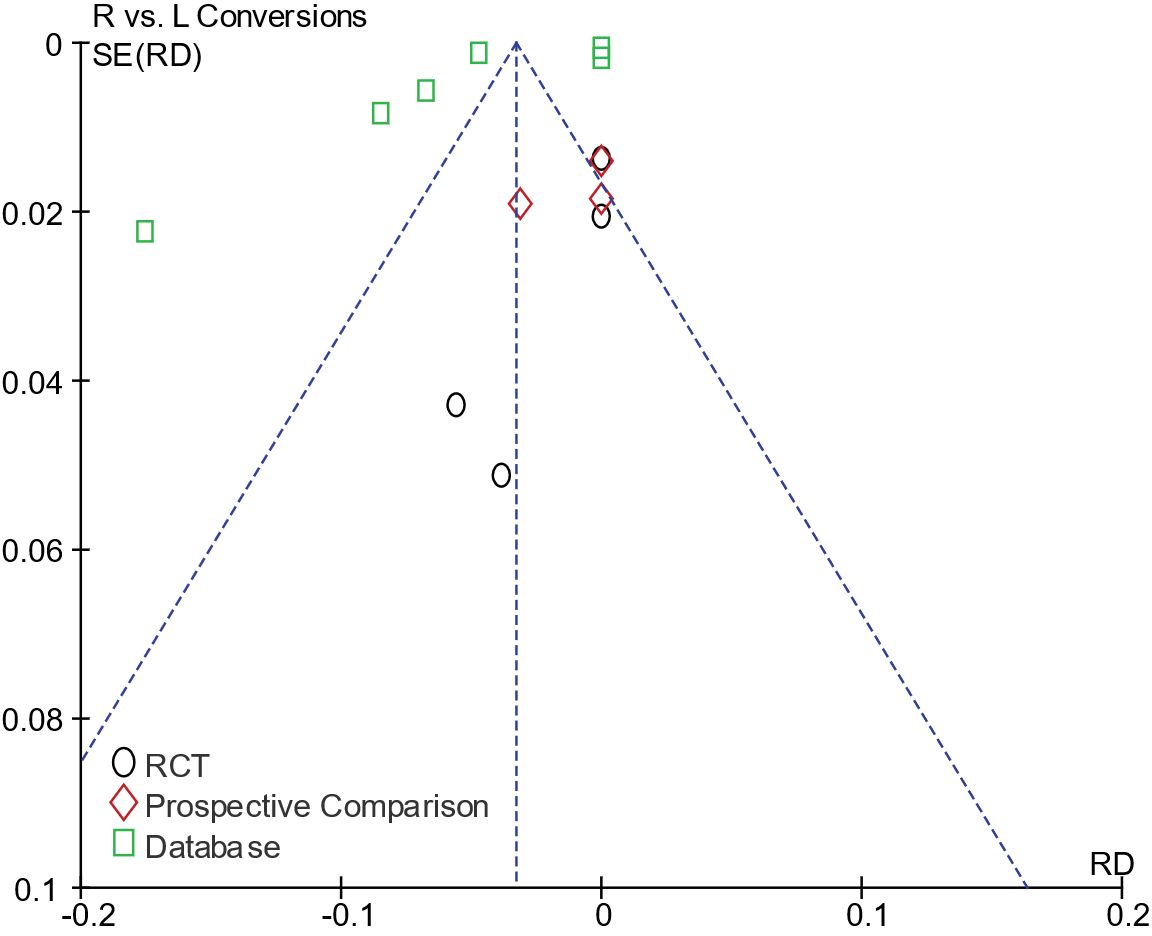

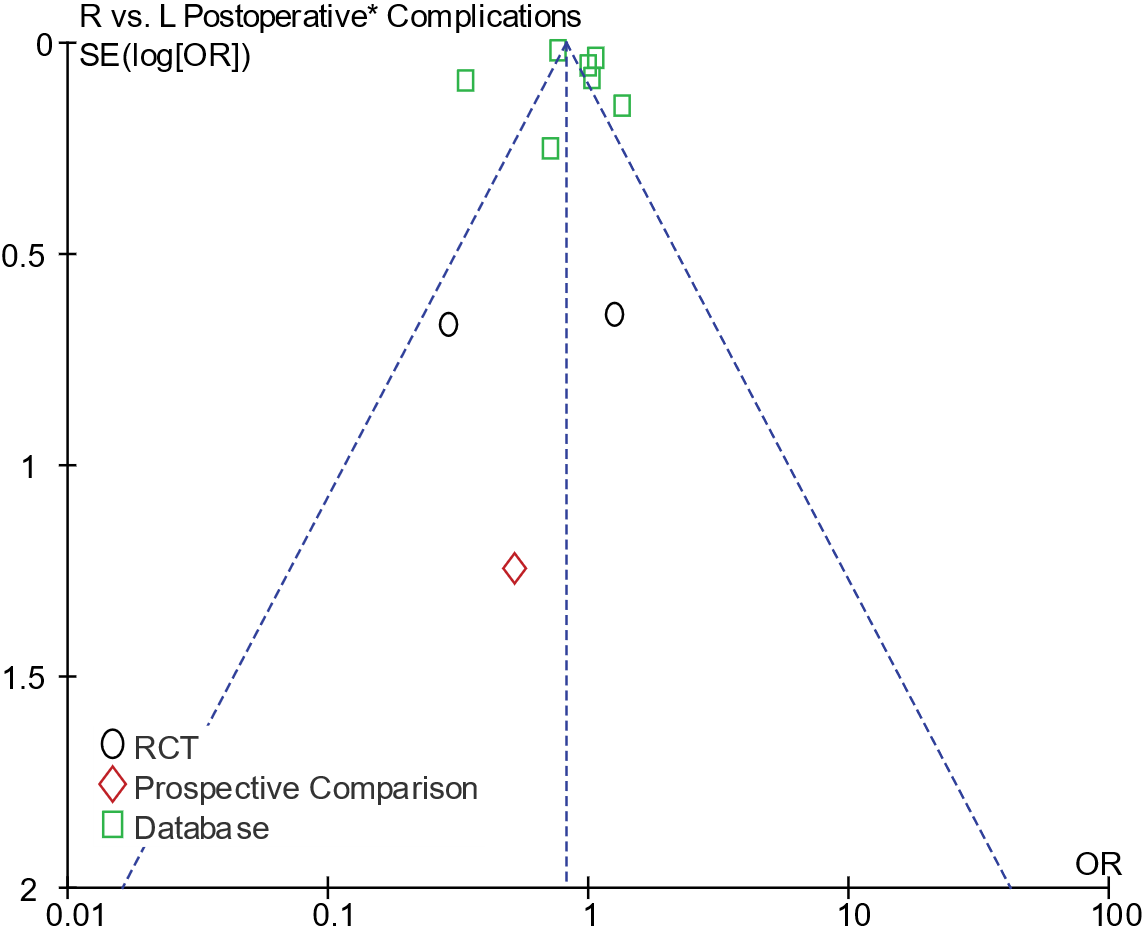


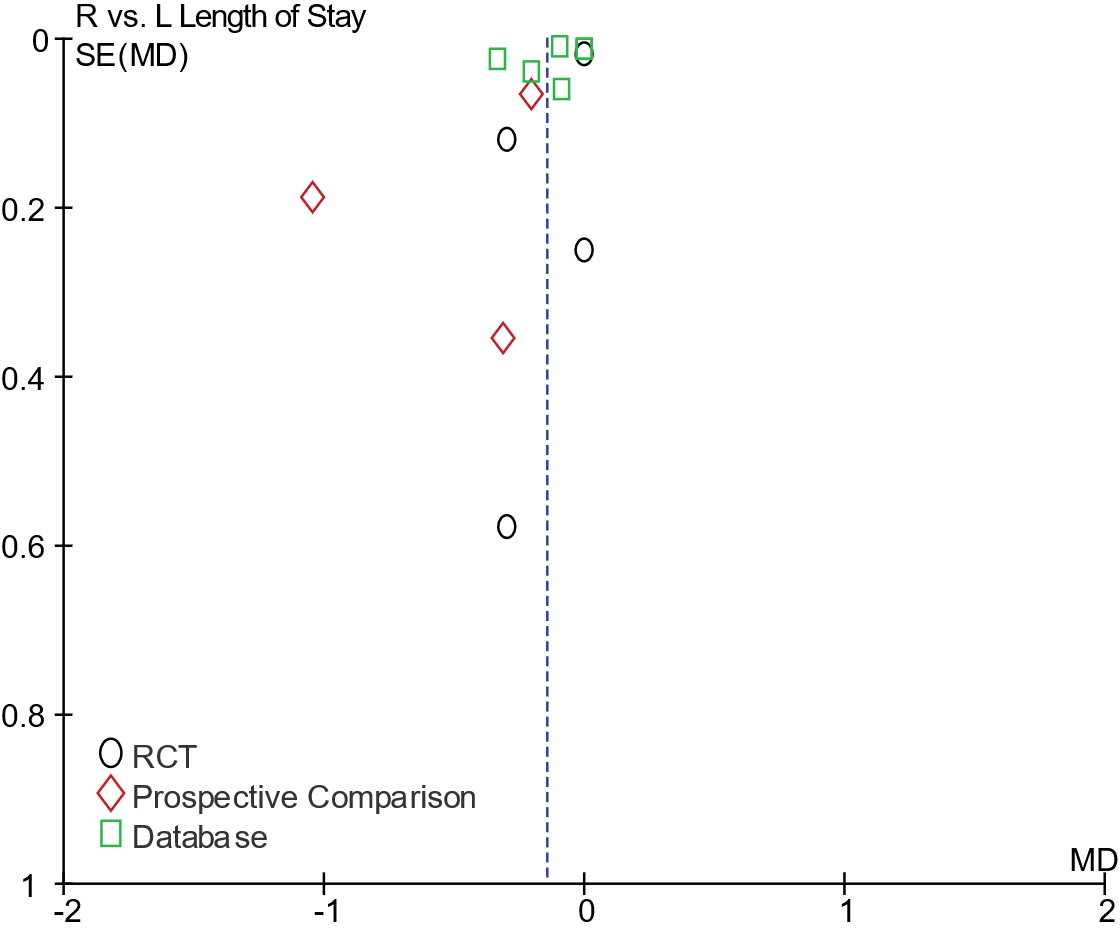

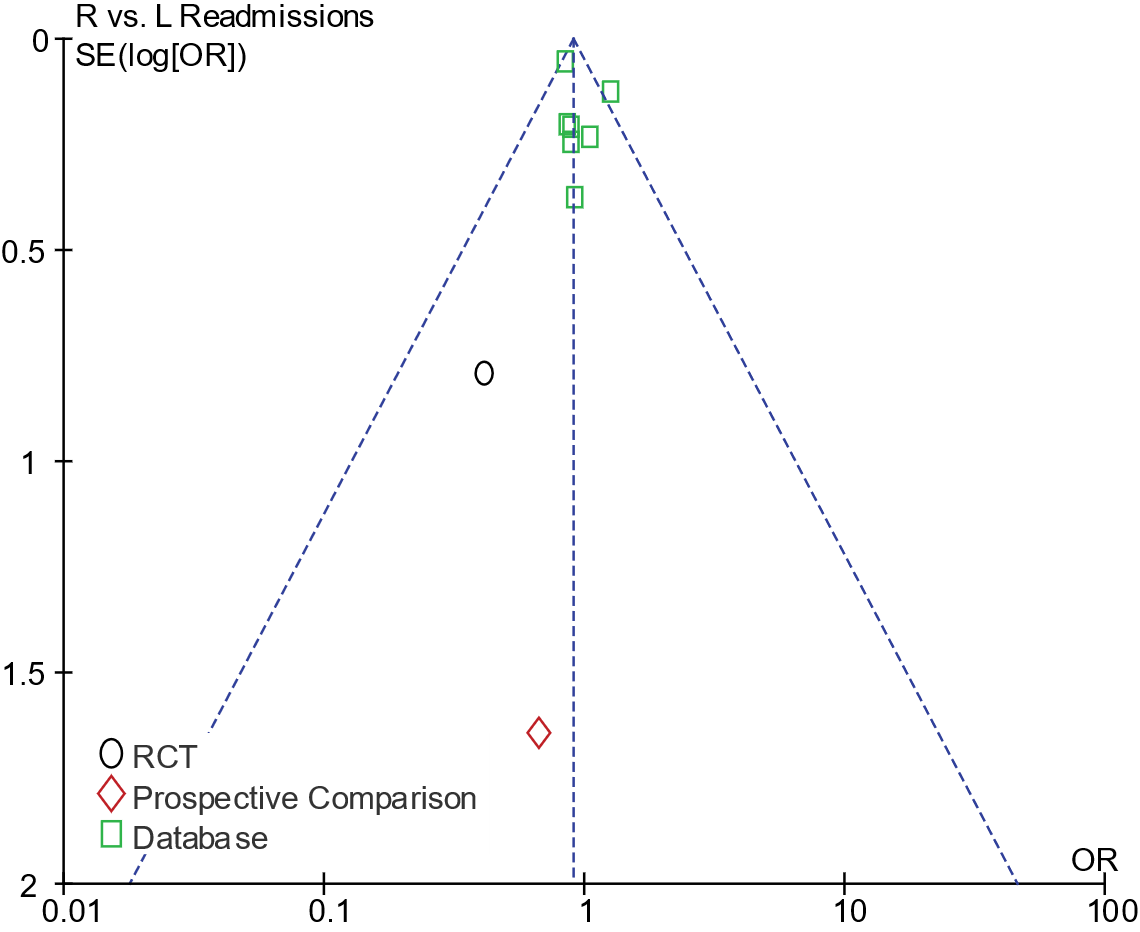

Supplement: Supplementary file 5 — Online Resource 5: Funnel Plots. Funnel plots for each outcome with at least 10 individual studies included in the analysis [file 11701_2023_1724_MOESM5_ESM.docx]
